# A prototype-aligned multi-scale attention network for few-shot industrial surface defect detection

**DOI:** 10.1371/journal.pone.0359400

**Published:** 2026-09-28

**Authors:** Rubing Huang, Lianyi Zhao

**Affiliations:** 1 School of Computer and Information Technology, Anhui Vocational and Technical College, Hefei, Anhui, China; 2 School of Electrical and Mechanical Engineering, Anhui Water Conservancy and Hydropower Vocational and Technical College, Hefei, Anhui, China; Sichuan University, CHINA

## Abstract

Few-shot industrial surface defect detection remains challenging because annotated defect images are scarce, defect scales vary considerably, and textured backgrounds can cause false responses. This study proposes a Prototype-Aligned Multi-Scale Attention Network (PA-MSAN) that integrates multi-receptive-field feature extraction, spatial-channel attention, and region of interest (RoI)-level prototype matching within a two-stage detector. Experiments are conducted on NEU-DET under 1-shot, 3-shot, and 5-shot episode settings, with 5-shot cross-domain evaluation on GC10-DET using target-domain support prototypes. Multi-scale feature-pyramid fusion represents local textures and broader defect morphology, while attention weighting enhances defect-sensitive regions and channels before region proposal network (RPN) proposal generation and RoI Align. Few-shot classification is performed by cosine similarity between RoI features and class prototypes, and training jointly optimizes detection classification, bounding-box regression, region proposal, and Prototype Alignment Loss (PAL). Under the 5-shot NEU-DET setting, PA-MSAN achieves 92.2 ± 0.4% mean average precision (mAP)@0.5, 88.7 ± 0.4% small-object average precision (APsmall), 0.89 ± 0.01 average intersection over union (IoU), and a 4.1 ± 0.2% background false response rate, with 23.4 ms detection time and 18.7 × 10⁶ parameters. Compared with Faster R-CNN, PA-MSAN improves mAP@0.5 by 2.9 percentage points and APsmall by 3.9 percentage points while reducing the background false response rate by 1.7 percentage points. Under the 5-shot target-domain support setting, PA-MSAN achieves 86.4 ± 0.4% mAP@0.5 on GC10-DET, 5.8 percentage points (6.3%) below the corresponding NEU-DET result. The results support region-level prototype alignment for limited-label metallic surface inspection under the evaluated datasets and two-stage configuration.

## 1. Introduction

Surface defect detection is an important part of ensuring product quality and production efficiency in industrial production. With the improvement of intelligent manufacturing and automation level, the demand for high-precision and real-time defect detection is increasing day by day. In complex industrial scenarios, there are numerous types of defects with significant differences in size and shape. At the same time, the background environment is complex, and traditional manual detection is not only inefficient, but also subject to subjective judgment errors [1]. Therefore, building intelligent systems that can adapt to diverse defect features and achieve reliable classification and detection under few-shot conditions has important academic value and application significance for improving the level of industrial automation [2].

In industrial production, surface defect detection is challenged by three closely related factors: limited annotated defect samples, large defect-scale variation, and strong background texture interference [3,4]. Scarce samples restrict deep networks from learning stable category-level representations, while scale variation requires multi-scale perception to capture both fine linear defects and larger defect regions [5,6]. In addition, small and irregular defect shapes together with mottled material textures can reduce localization accuracy if the detector relies only on single-level features or shallow feature representations [7,8].

Existing studies address these challenges from complementary directions. Convolutional neural network (CNN)-based detectors provide a basic framework for automated defect localization but remain sensitive to scarce samples and small defects [9,10]. Multi-scale feature fusion and feature-pyramid designs improve sensitivity to defects of different sizes [11,12], while recent few-shot industrial detectors use bidirectional feature fusion and multi-stage proposal refinement to improve localization under limited supervision [13]. Attention-based methods strengthen defect-related responses and suppress background textures [14,15], and attention-guided few-shot frameworks further use channel-spatial weighting and support-query semantic alignment [16]. Prototype learning aggregates scarce class features into compact representations and improves inter-class separability [17,18]. Representative few-shot detectors adopt different transfer mechanisms: TFA performs classifier-level fine-tuning [19], FSRW reweights query features using support information [20], and Meta R-CNN introduces RoI-level meta features for low-shot detection [21]. PA-MSAN differs by constructing explicit class prototypes from attention-enhanced support RoI features, comparing RoI embeddings with those prototypes through cosine similarity, and regularizing the shared RoI embedding with Prototype Alignment Loss.

This study formulates few-shot industrial surface defect detection as a region-level prototype-alignment problem. PA-MSAN first constructs multi-receptive-field pyramid features to preserve defect information across scales and then applies spatial-channel attention to the fused representation before proposal generation. Support-region features obtained after RoI Align are aggregated into class prototypes, and RoI features are matched to those prototypes in the same cosine-similarity space. RPN proposal learning and bounding-box regression optimize localization, while prototype matching and Prototype Alignment Loss optimize few-shot class discrimination on the shared RoI representation. The resulting architecture links multi-scale representation, attention refinement, region localization, and metric-based classification within one training objective. Table 1 isolates the contributions of the multi-scale, attention, prototype-learning, and PAL components.

**Table 1:** Quantitative ablation results under the 5-shot NEU-DET setting.

Variant	mAP@0.5 Mean ± Std (%)	mAP drop compared with full model (percentage points)	Main interpretation
Full PA-MSAN	92.2 ± 0.4	—	Highest mAP among evaluated variants
w/o MSFE	81.5 ± 0.3	10.7	Removing MSFE produces the largest mAP decrease among the evaluated ablations
w/o PL	84.3 ± 0.3	7.9	Removing the prototype-learning branch lowers few-shot classification performance
w/o PAL	88.9 ± 0.4	3.3	Removing PAL while retaining the prototype classifier lowers mAP by 3.3 points
w/o SA	87.2 ± 0.3	5.0	Removing spatial attention lowers mAP by 5.0 points
w/o CA	88.4 ± 0.3	3.8	Removing channel attention lowers mAP by 3.8 points

Note: mAP drops are computed from the mean mAP@0.5 values, with the complete PA-MSAN used as the reference baseline. Values are reported as mean ± standard deviation over five repeated runs using seeds 0–4.

**The main contributions of this paper are as follows:**

(1) PA-MSAN is proposed to formulate few-shot industrial surface defect detection as an RoI-level prototype-alignment task. Support-set prototypes are constructed from attention-enhanced region features and directly guide RoI classification. Localization is optimized through the shared RoI representation together with RPN and bounding-box regression objectives, thereby coupling region-level metric learning with two-stage detection.
(2) A hierarchical feature representation module is developed for the defect detection task by combining feature-pyramid fusion with convolutions of multiple receptive fields. With this design, the detector can describe subtle local textures, medium-scale defect structures, and higher-level semantic information even when only a few support samples are available.
(3) A defect-sensitive prototype matching strategy is introduced at the RoI level by using spatial-channel attention together with RoI Align. The attention mechanism helps filter out irrelevant background textures, and the cosine similarity between region features and class prototypes allows the detector to distinguish defect categories more effectively when only a few labeled examples are available.
(4) A joint training objective is designed by optimizing detection classification loss, bounding-box regression loss, RPN loss, and Prototype Alignment Loss together. Through this objective, the model learns more accurate region localization and more discriminative feature distributions, with samples from the same class becoming closer and different classes remaining better separated. This helps improve the stability of few-shot detection and supports cross-domain adaptation with limited target-domain supervision.

## 2. Network design and method implementation

This paper proposes PA-MSAN for surface defect detection with scarce labeled samples and substantial scale variation. The network contains three interacting components: multi-scale feature extraction, attention enhancement, and prototype-based region classification, which are optimized jointly with the two-stage detection objectives. Fig 1 summarizes the overall processing flow.

**Fig 1:**
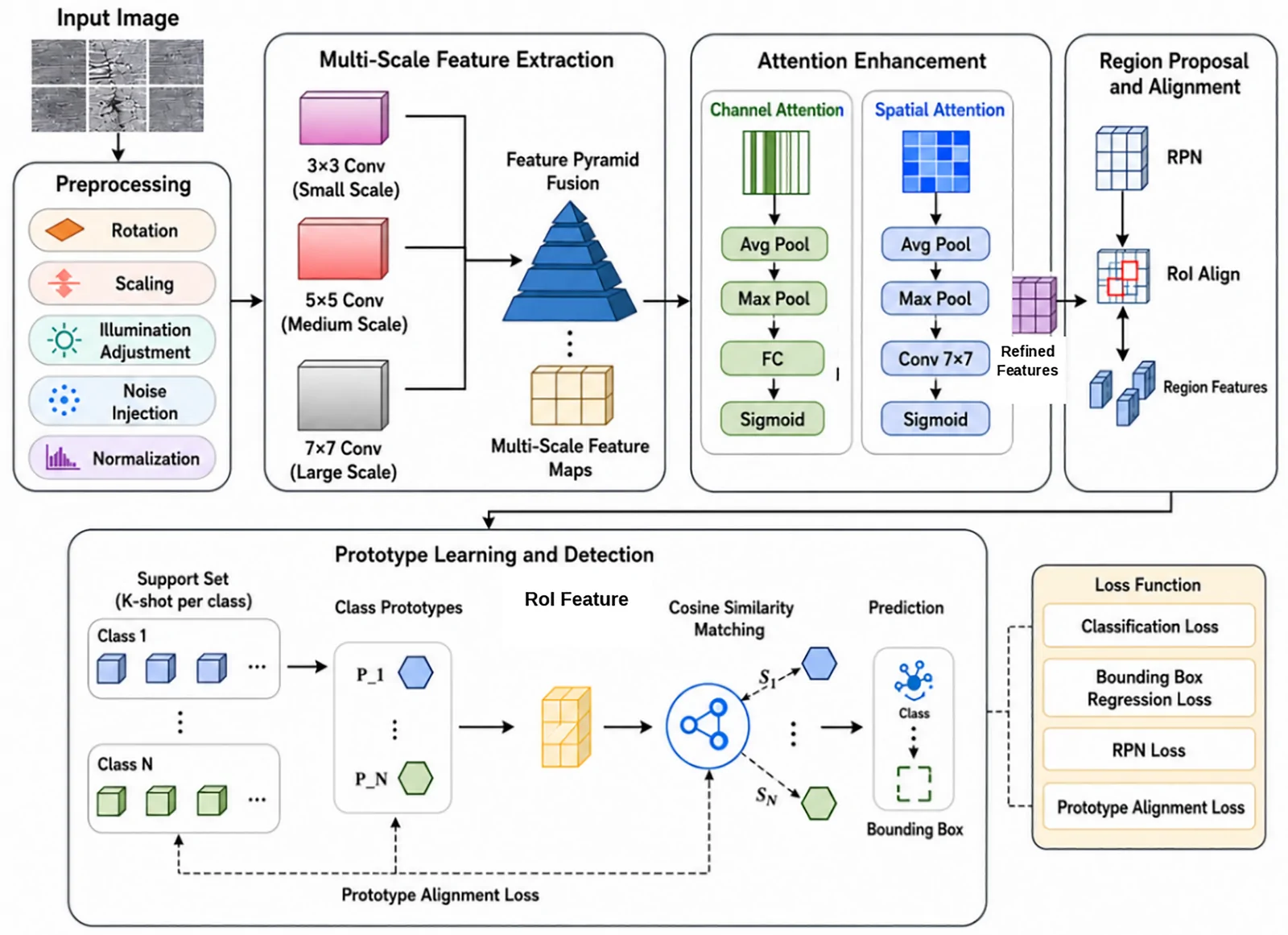
Overall framework of PA-MSAN. Source material, where applicable: Reprinted from the NEU-DET dataset [22,23] under a CC BY 4.0 license, with permission from Kechen Song, original copyright 2013. Feature visualizations, annotations, and figure composition were generated by the authors.

Fig 1 shows the complete processing flow of the proposed method from input image to defect classification. The input image is first preprocessed and normalized, and is then processed by a multi-scale feature extraction (MSFE) module to generate hierarchical features under different receptive fields. Then, the multi-scale attention mechanism applies adaptive weights to spatially significant regions and key channels, and outputs attention-enhanced features. The RPN then generates candidate defect regions from the enhanced features, and region features are extracted through RoI Align. Finally, cosine similarity matching is performed with prototype vectors of various categories in the prototype learning module, combined with bounding-box regression and PAL to complete defect detection and localization under few-shot conditions.

### 2.1. Data augmentation and preprocessing

Industrial surface images are affected by illumination variation, background texture, and sensor noise, which can further limit feature learning when only a few labeled defect images are available [24]. The preprocessing pipeline therefore combines geometric and photometric augmentation with consistent grayscale normalization while preserving the annotated defect structure [25, 26]. NEU-DET [22,23] contains 200 × 200 grayscale steel-surface images, whereas GC10-DET [27] contains grayscale metallic surface-defect images with variable resolutions in commonly used releases. All images are read or converted as single-channel grayscale inputs, resized to 416 × 416, and normalized to the 0–1 range. During training, rotation, flipping, illumination perturbation, scaling, and Gaussian noise are applied according to the ranges in Table 2; validation and test images use deterministic resizing and normalization only.

**Table 2:** Data augmentation parameter settings.

Augmentation Type	Symbol	Range	Description
Rotation	*θ*_*r*_	[−30°, 30°]	Rotation angle
Scaling	*θ*_*s*_	[0.8, 1.2]	Scale factor
Illumination Gain	α	[0.8, 1.2]	Pixel gain factor
Illumination Offset	β	[−10, 10]	Intensity offset applied before [0,255] clipping and min-max normalization
Gaussian Noise	*σ*_*n*_	0.0–0.05	Noise standard deviation applied after min-max normalization in the 0–1 intensity space

The geometric and photometric augmentation sequence is defined as follows:


$$\begin{array}{c} I^{\prime }=\text{clip}(A_{\text{photo}}\left(A_{\text{geo}}\left(I;\theta_{r},\theta_{s}\right);\alpha ,\beta \right),\text{0},\text{255}) \end{array}$$ (1)

In Equation (1), I denotes the original image, $I^{\prime }$ denotes the clipped geometrically and photometrically augmented image, $A_{geo}$(·) includes rotation, scaling, and horizontal/vertical flipping, and $A_{photo}$(·) applies illumination gain and offset. The continuous parameters $\theta_{r}$, $\theta_{s}$, α, and β follow the ranges in Table 2. The illumination-adjusted intensities are saturated to the [0,255] grayscale range in Equation (1) before min-max normalization.

The clipped photometrically augmented image is min-max normalized before Gaussian noise is added in the normalized 0–1 intensity space:


$$\begin{array}{c} I_{\text{norm}}=\frac{I^{\prime }-I_{\text{min}}}{I_{\text{max}}-I_{\text{min}}},\;\;\tilde{I}=\text{clip}\left(I_{\text{norm}}+\epsilon_{n},\text{0},\text{1}\right),\;\epsilon_{n}\sim N\left(\text{0},\sigma_{n}^{\text{2}}\right) \end{array}$$ (2)

In Equation (2), $I_{norm}$ is the min-max normalized image, $\varepsilon_{n}$ is zero-mean Gaussian noise with standard deviation $\sigma_{n}$, and the final input is clipped to the 0–1 range. This ordering keeps the illumination transformation before normalization and defines the Gaussian-noise magnitude in the same normalized intensity space used in Table 2. The minimum and maximum intensities used for min-max normalization are computed from the clipped output of Equation (1).

Each defect class is augmented after the 70%/15%/15% dataset split. Geometric augmentation applies rotation, horizontal and vertical flipping, and scaling together with the corresponding bounding-box transformation. Illumination gain and offset are then applied and the resulting intensities are clipped to the [0,255] grayscale range before min-max normalization, followed by Gaussian noise in the normalized 0–1 intensity space. Every augmented view inherits the subset assignment of its source image, so transformations derived from one original image remain within the same training, validation, or test subset.

Fig 2 shows some typical original and augmented industrial surface-defect samples. Augmentation operations include rotation, flipping, scaling, illumination gain and offset, and Gaussian noise injection; at the same time, the main defect texture and contour structure are retained for follow-up multi-scale feature extraction.

**Fig 2:**
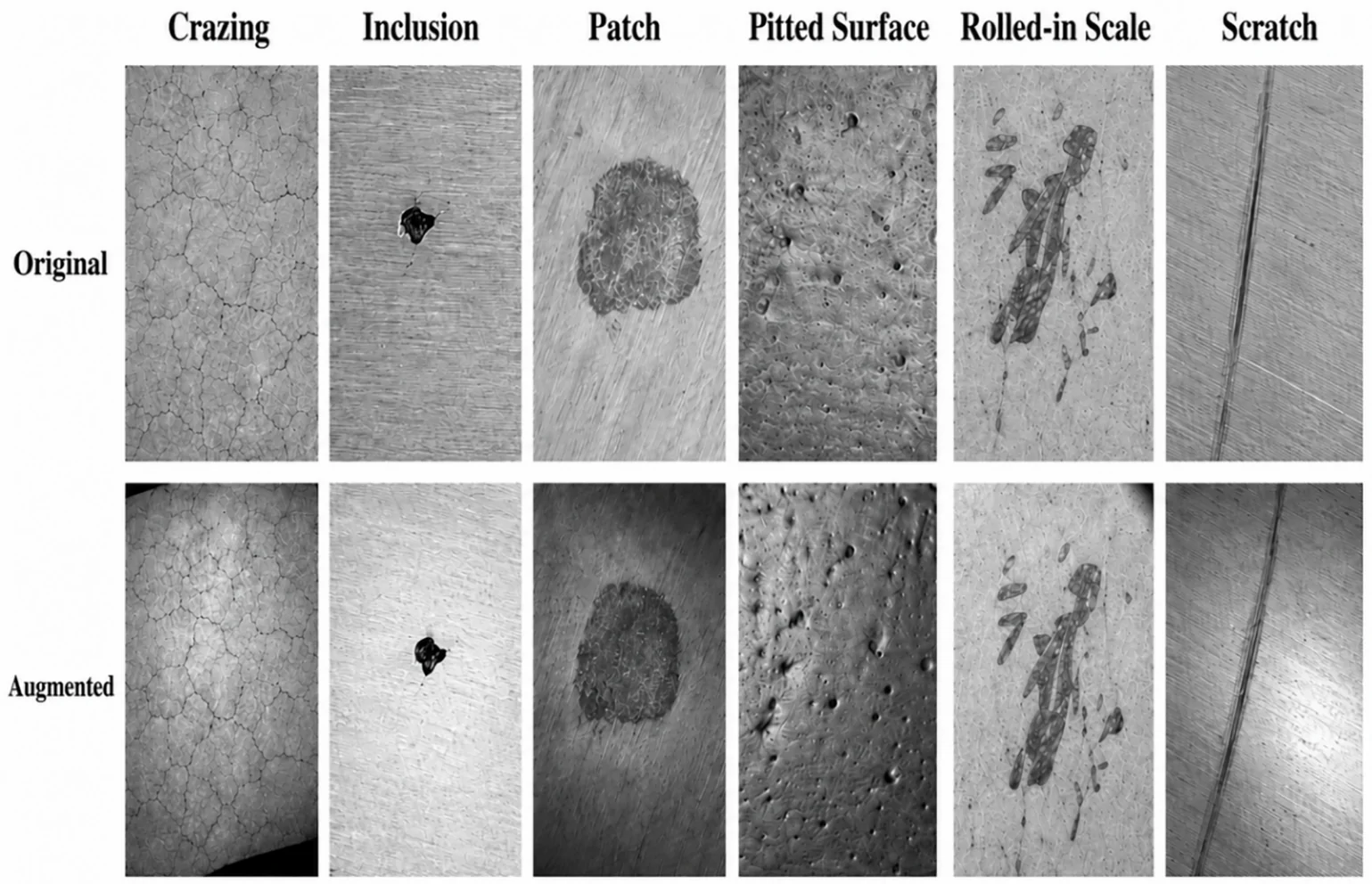
Representative augmented samples of industrial surface defects. Source material, where applicable: Reprinted from the NEU-DET dataset [22,23] under a CC BY 4.0 license, with permission from Kechen Song, original copyright 2013. Feature visualizations, annotations, and figure composition were generated by the authors.

As shown in Fig 2, the upper row contains original defect samples and the lower row shows their augmented counterparts. These transformations simulate changes in viewing geometry, illumination, and sensor noise while retaining the defect texture and contour needed for subsequent feature extraction and prototype learning.

A unified set of data-augmentation parameters is used, and the specific numerical ranges of rotation angle, scale ratio, lighting gain coefficient, lighting offset and Gaussian noise standard deviation are listed in Table 2.

The parameters in Table 2 are sampled from continuous intervals for rotation, scaling, illumination, and Gaussian noise. Illumination gain and offset are applied, and the resulting intensities are clipped to [0,255], before min-max normalization, and Gaussian noise is added afterward in normalized intensity units. Random augmentation is restricted to support-image training views; validation and test images undergo deterministic resizing to 416 × 416 and min-max normalization. Transformed versions of the same source image remain within a single data subset.

### 2.2. Multi-scale feature extraction module

To represent industrial defects with substantial scale variation, PA-MSAN combines multi-receptive-field convolution with a feature-pyramid pathway containing top-down fusion and lateral connections [28–30]. The module produces hierarchical feature maps that preserve fine texture details, intermediate defect structures, and broader semantic context [31,32]. Lateral projections align channel dimensions before feature addition, and the resulting pyramid features provide multi-scale inputs for attention refinement, proposal generation, prototype matching, classification, and box regression.

The convolution operation can be expressed as:


$$\begin{array}{c} F^{\left(l\right)}=\delta \left(W^{\left(l\right)}*F^{\left(l-\text{1}\right)}+b^{\left(l\right)}\right) \end{array}$$ (3)

In Equation (3), *F*^*(l)*^ denotes the feature map of the *l*-th convolutional layer, *F*^*(l−1)*^ denotes the input feature map from the previous layer, *W*^*(l)*^ is the convolution kernel, *b*^*(l)*^ is the bias term, * denotes convolution, and δ(·) denotes the rectified linear unit (ReLU) activation function. Convolution kernels of 3 × 3, 5 × 5, and 7 × 7 are used at different levels to balance the responses to small and large defects [33,34].

Multi-scale features are integrated through the feature-pyramid fusion mechanism. To make element-wise fusion dimensionally valid when adjacent feature levels have different channel numbers, the lateral feature is first projected to the target channel dimension:


$$\begin{array}{c} P_{l}=\phi_{l}\left(F_{l}\right)+\text{Up}\left(P_{l+\text{1}}\right) \end{array}$$ (4)

In Equation (4), $P_{l}$ denotes the fused pyramid feature at level l, $F_{l}$ denotes the lateral feature at level l, $\phi_{l}$(·) denotes a lateral projection, typically implemented by a 1 × 1 convolution, and *Up*(·) denotes the upsampling operation used to align spatial resolution. This projection avoids channel-dimensional mismatch before feature addition.


$$\begin{array}{c} D_{l-\text{1}}=\text{Down}\left(P_{l}\right) \end{array}$$ (5)

In Equation (5), *D*_*l−1*_ denotes the downsampled feature map and Down(·) denotes the downsampling operation with a stride of 2. Thus, it can build a multi-scale feature pyramid and maintain spatial consistency among the feature maps at different scales [35,36].

Fig 3 shows the response of the multi-scale feature extraction module. Fine-scale features retain local edge and texture details; mid-scale features are structural defect patterns; and global feature maps focus on large-scale semantic regions. The above responses serve as hierarchical inputs for the following attention and prototype-learning modules.

**Fig 3:**
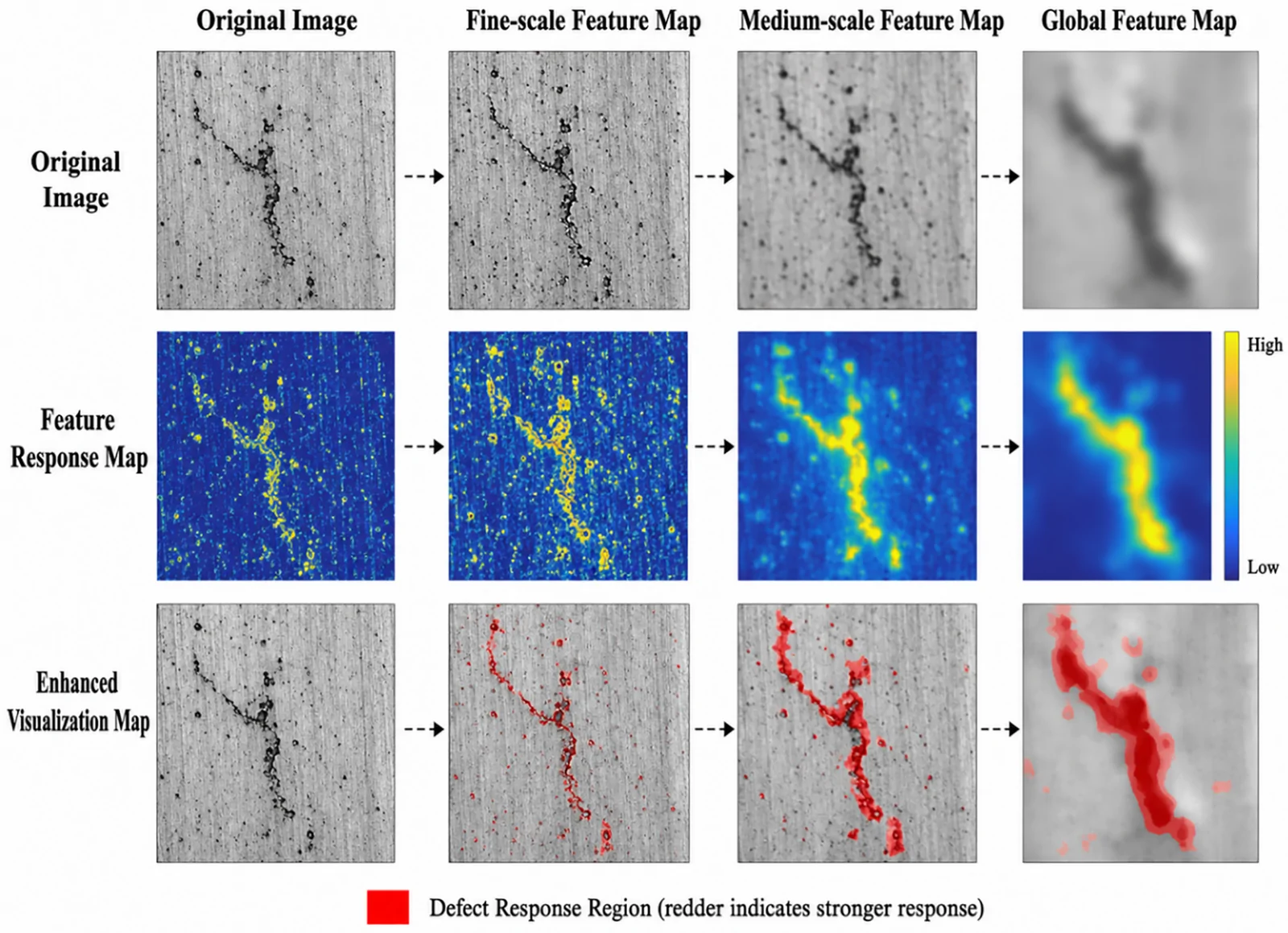
Multi-scale feature response visualization of industrial defects. Source material, where applicable: Reprinted from the NEU-DET dataset [22,23] under a CC BY 4.0 license, with permission from Kechen Song, original copyright 2013. Feature visualizations, annotations, and figure composition were generated by the authors.

As shown in Fig 3, the normalized input is converted into fine-grained, medium-grained and global feature representations. High-response defect regions are more concentrated at higher levels of the hierarchy, and multi-scale convolution and feature-pyramid fusion improve the feature representation by incorporating local texture details and global defect morphology.

Table 3 summarizes the kernel sizes, output channels, and implementation-level computational costs of the multi-scale blocks. Explicit 5 × 5 and 7 × 7 kernels provide direct medium- and large-receptive-field observations. Table 4 compares this multi-kernel configuration with a stacked-3 × 3 variant under matched channel settings and the same feature pyramid network (FPN), attention, prototype branch, training budget, and evaluation protocol. The proposed configuration improves mAP@0.5 by 1.8 percentage points and APsmall by 2.8 percentage points while using comparable feature-extractor parameters and lower reported floating-point operations (FLOPs).

**Table 3:** Multi-scale convolutional kernel and channel configuration.

Layer	Kernel Size	Output Channels	Function	Params (K)	FLOPs (M)
Layer 1	3 × 3	64	Fine texture extraction	0.6	3.4
Layer 2	5 × 5	128	Mid-scale feature extraction	204.8	38.9
Layer 3	7 × 7	256	Large-scale global feature extraction	1605.6	96.5
Layer 4	3 × 3	256	Fine–mid feature fusion	589.8	61.2
Layer 5	5 × 5	512	Mid–large feature fusion	1638.4	118.7

Note: The reported Params and FLOPs cover the profiled convolutional blocks in the multi-scale/FPN pathway for single-channel 416 × 416 input. Lateral projections and feature-fusion operations are profiled using their implementation-level tensor dimensions.

**Table 4:** Comparison between a stacked 3 × 3 convolutional variant and the proposed multi-kernel (3 × 3/5 × 5/7 × 7) design under the 5-shot NEU-DET setting.

Variant	mAP@0.5 Mean ± Std (%)	APsmall (%)	Feature-extractor Params (M)	Feature-extractor FLOPs (G)	Training budget (epochs)
Stacked 3 × 3 (matched channel configuration)	90.4 ± 0.4	85.9 ± 0.5	3.8	27.9	200
Proposed multi-kernel design (PA-MSAN)	92.2 ± 0.4	88.7 ± 0.4	4.0	25.7	200

Note: Both variants share the same FPN, attention modules, prototype branch, and training protocol (seeds 0–4, 200 epochs, Adam with an initial learning rate of 1 × 10^−3^). Values are reported as mean ± standard deviation over seeds 0–4. The Params and FLOPs columns in Table 4 refer to the feature-extraction configurations evaluated in this comparison. The 18.7 M total-parameter count corresponds to the complete detector, including the RPN, RoI head, attention modules, and prototype-related components.

Table 3 defines the kernel sizes and channel allocation used in the multi-scale feature extractor. The resulting pyramid features provide inputs to the attention module and the downstream proposal, RoI, prototype-classification, and box-regression stages.

For FPN fusion, lateral features from different levels are projected to a common feature dimension before element-wise addition, consistent with the projection operation defined in Equation (4).

### 2.3. Attention mechanism and feature enhancement

The hierarchical feature map generated by the multi-scale feature extraction module contains local and global information for defects of different sizes. The attention module applies channel weighting first and spatial weighting second to the fused pyramid feature. The input $F_{p}$ is used to generate channel weights $M_{c}\left(F_{p}\right)$; channel-weighted features $F_{c}$ are then used to generate spatial weights $M_{s}\left(F_{c}\right)$. The resulting attention-enhanced feature $F_{a}$ is shared by the RPN, RoI Align, prototype branch, classification head, and box-regression branch [37,38].

Channel attention uses global average pooling and global maximum pooling to summarize the fused pyramid feature and maps the resulting descriptors through a two-layer fully connected network:


$$\begin{array}{c} M_{c}\left(F_{p}\right)=\sigma \left(W_{\text{2}}\delta \left(W_{\text{1}}\text{GA}\text{P}\left(F_{p}\right)\right)+W_{\text{2}}\delta \left(W_{\text{1}}\text{GMP}\left(F_{p}\right)\right)\right) \end{array}$$ (6)

In Equation (6), *M*_*c*_(*F*_*p*_) denotes the channel attention weight generated from the pyramid feature *F*_*p*_. Global average pooling (GAP) and global maximum pooling (GMP) are denoted by GAP(·) and GMP(·), respectively; *W*_*1*_ and *W*_*2*_ are the weight matrices of the two fully connected layers, δ(·) is the ReLU activation function, and σ(·) is the Sigmoid function. Channel attention strengthens defect-related channels and suppresses background-noise channels.

After channel weighting, spatial attention averages and maximizes $F_{c}$ along the channel dimension and applies a 7 × 7 convolution to generate the spatial attention map [39]:


$$\begin{array}{c} M_{s}\left(F_{c}\right)=\sigma \left(f^{\text{7}\times \text{7}}\left(\left[\text{AvgPool}\left(F_{c}\right);\text{MaxPool}\left(F_{c}\right)\right]\right)\right) \end{array}$$ (7)

In Equation (7), $M_{s}\left(F_{c}\right)$ denotes the spatial attention weight generated from $F_{c}$, [·;·] denotes channel-wise concatenation, $f^{\text{7}\times \text{7}}$(·) denotes a 7 × 7 convolution, and AvgPool(·) and MaxPool(·) denote channel-wise average and maximum pooling, respectively [40].

Feature weighting combines channel and spatial attention to generate the final enhanced feature Fₐ, which is represented as:


$$\begin{array}{c} F_{c}=F_{p}⊙M_{c}\left(F_{p}\right),\;\;F_{a}=F_{c}⊙M_{s}\left(F_{c}\right) \end{array}$$ (8)

In Equation (8), $F_{c}$ is the channel-weighted intermediate feature and $F_{a}$ is the final channel-spatial attention output. The sequential formulation makes the spatial mask depend on the channel-refined representation and matches the channel-to-spatial processing flow used in Fig 4.

**Fig 4:**
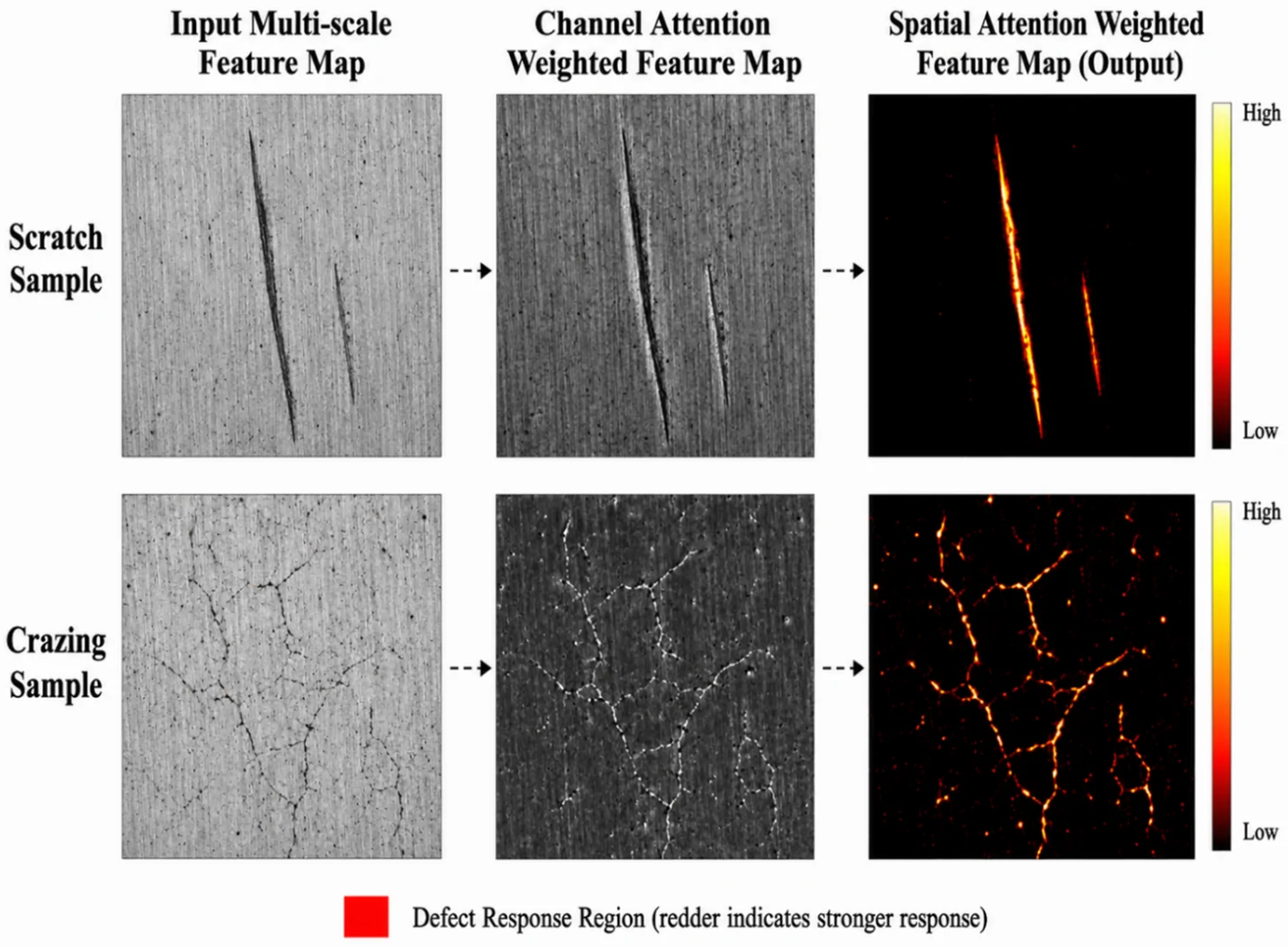
Qualitative feature-response visualization of the attention enhancement module. Source material, where applicable: Reprinted from the NEU-DET dataset [22,23] under a CC BY 4.0 license, with permission from Kechen Song, original copyright 2013. Feature visualizations, annotations, and figure composition were generated by the authors.

Fig 4 is the qualitative feature-response visualization after attention enhancement. Warmer or brighter regions indicate stronger defect-related responses, showing that the attention module strengthens defect-sensitive regions while suppressing irrelevant background texture responses.

The attention enhancement module performs channel and spatial weighting on the input multi-scale feature map and outputs defect-sensitive feature responses. The qualitative visualization indicates that small Crazing and Scratch regions are more clearly reinforced in complex backgrounds, supporting subsequent RPN proposal generation, RoI Align feature extraction, and detection-head localization.

### 2.4. Prototype learning and few-shot classification

Under few-shot conditions, a detector must distinguish defect categories from a small number of labeled support images. PA-MSAN constructs class representations from fixed-size RoI features at the region level. Candidate regions are aligned through RoI Align, projected to the prototype embedding space, and aggregated by class. For each class k, the prototype p_k_ is the arithmetic mean of its support-region features and is defined as:


$$\begin{array}{c} p_{k}=\frac{\text{1}}{\left|S_{k}\right|}\sum f_{i}\in S_{k}\;f_{i} \end{array}$$ (9)

In Equation (9), $p_{k}$ denotes the prototype vector of category *k*, $S_{k}$ denotes the support feature set of category *k*, |Sk| is the number of support samples in that category, and $f_{i}$ denotes the region feature vector extracted by RoI Align. RoI Align reduces boundary quantization errors and improves prototype consistency for small defects by producing fixed-size region representations before projection into the embedding space.

The matching between features and prototypes is measured using cosine similarity, defined as:


$$\begin{array}{c} s_{i,k}=\frac{f_{i}^{\text{ T}}p_{k}}{∥f_{i}{∥}_{\text{2}}∥p_{k}{∥}_{\text{2}}} \end{array}$$ (10)

In Equation (10), *s*_*i,k*_ ∈ [−1, 1] denotes the cosine similarity between the input region feature *f*_*i*_ and the class prototype *p*_*k*_, ‖·‖_2_ denotes the L2 norm, and *f*_*i*_^T^*p*_*k*_ denotes the inner product. The similarity distribution over all class prototypes is used for few-shot defect classification.

The cosine-similarity scores are converted to class probabilities by a softmax over the K defect prototypes:


$$\begin{array}{c} P\left(y_{i}=k|f_{i}\right)=\frac{\text{exp}\left(s_{i,k}\right)}{\sum_{j=\text{1}}^{K}\text{exp}\left(s_{i,j}\right)} \end{array}$$ (11)

In Equation (11), the cosine-similarity scores are normalized across the K defect prototypes to obtain the prototype-based class probability for each RoI feature. The resulting probability distribution is used for few-shot class prediction and in the Prototype Alignment Loss.

During optimization, PAL increases the similarity of each labeled training RoI embedding derived from the K-shot support images and their augmented views to its ground-truth class prototype while suppressing similarities to competing prototypes [41,42]. Related few-shot metal-defect work has explored adaptive support-feature correlation and hierarchical multi-scale fusion for cross-domain generalization [43].


$$\begin{array}{c} L_{\text{PAL}}=-\frac{\text{1}}{N}\sum_{i=\text{1}}^{N}\text{log}P\left(y_{i}|f_{i}\right) \end{array}$$ (12)

In Equation (12), $L_{\text{PAL}}$ denotes the Prototype Alignment Loss, N is the number of labeled training RoI embeddings included in the loss, and $y_{i}$ is the ground-truth class of embedding $f_{i}$. The loss is computed from RoIs derived from the K-shot support images and their augmented training views.

Fig 5 visualizes prototype-learning effects by projecting high-dimensional region features into a two-dimensional space using t-distributed stochastic neighbor embedding (t-SNE). Different colors denote different defect categories, and the cosine-similarity heatmap summarizes the similarity relationship among class prototypes.

**Fig 5:**
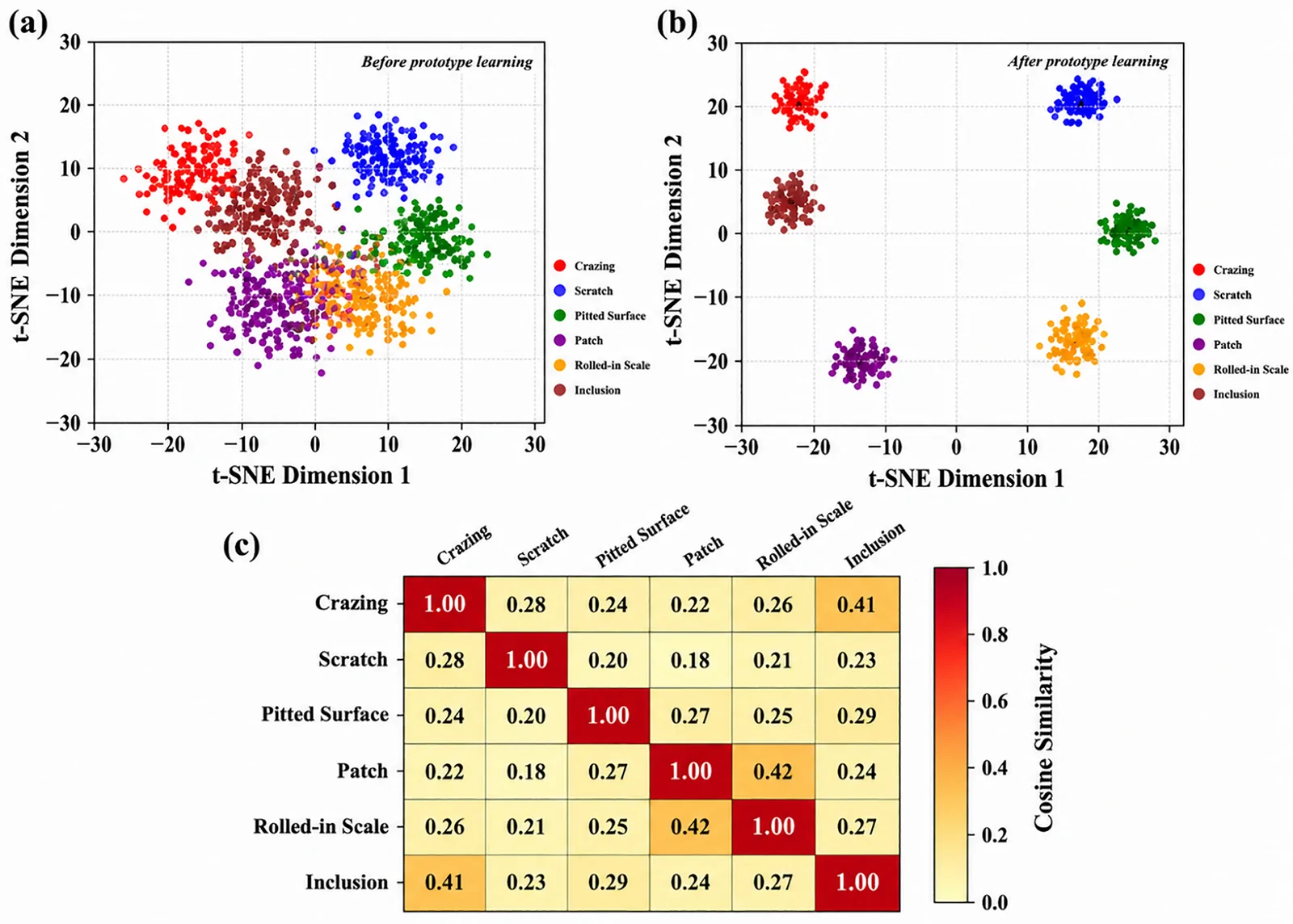
Feature distribution and prototype-similarity analysis under prototype learning. (a) Attention-enhanced feature distribution without prototype learning. (b) Feature clustering after introducing prototype learning. (c) Prototype cosine-similarity heatmap.

Fig 5 compares attention-enhanced RoI features before and after prototype learning. The projected features become more compact within classes and more separated between classes after prototype alignment, and the prototype cosine-similarity heatmap summarizes the remaining inter-class relationships. In PA-MSAN, the prototypes are constructed from RoI-Aligned support-region features, so the metric representation is defined at the same region level used by the detection head.

### 2.5. Joint optimization and training strategy

PA-MSAN uses a joint objective to optimize region classification, box regression, proposal generation, and prototype-space structure within the same training procedure. The total loss is defined as:


$$\begin{array}{c} L_{\text{joint}}=\lambda_{d}L_{\text{det}}+\lambda_{r}L_{\text{reg}}+\lambda_{\text{RPN}}L_{\text{RPN}}+\lambda_{p}L_{\text{PAL}} \end{array}$$ (13)

In Equation (13), $L_{\text{joint}}$ denotes the total joint loss, $L_{\text{det}}$ is the detection classification loss, $L_{\text{reg}}$ is the bounding-box regression loss, $L_{\text{RPN}}$ is the region proposal loss, and $L_{\text{PAL}}$ is the Prototype Alignment Loss. The coefficients $\lambda_{d}$, $\lambda_{r}$, $\lambda_{\text{RPN}}$, and $\lambda_{p}$ weight the four objectives.

The detection classification loss $L_{\text{det}}$ is cross-entropy on the linear classification logits, $L_{\text{reg}}$ is Smooth L1 loss for bounding-box regression, and $L_{\text{RPN}}$ combines RPN objectness and proposal-regression losses. $L_{\text{PAL}}$ structures the prototype embedding while sharing the upstream RoI representation with the auxiliary linear classifier. Table 1 separates the complete prototype-learning branch from PAL: removing the full prototype-learning branch lowers mAP@0.5 by 7.9 percentage points, while removing PAL with the prototype classifier retained lowers mAP@0.5 by 3.3 points. During inference, the prototype metric head provides the few-shot class decision and the linear classification head remains an auxiliary training branch.

The detection classification head computes linear classification logits from the RoI feature as:


$$\begin{array}{c} z_{i}=Wf_{i}+b \end{array}$$ (14)

In Equation (14), $f_{i}$ denotes the RoI feature vector for candidate region i, W and b are the learnable parameters of the auxiliary linear classification head, and $z_{i}$ contains the logits supervised by $L_{det}$. The prototype metric head uses Equations (10) and (11) to compute cosine similarities and normalized prototype class probabilities.

The network parameters are updated using Adam optimizer, and the optimization formula is as follows:


$$\begin{array}{c} \theta_{t+\text{1}}=\theta_{t}-\eta \frac{{\hat{m}}_{t}}{\sqrt{{\hat{v}}_{t}}+\epsilon } \end{array}$$ (15)

In Equation (15), $\theta_{t}$ denotes the network parameters at iteration t, η is the learning rate, ${\hat{m}}_{t}$ and ${\hat{v}}_{t}$ are the bias-corrected first- and second-moment estimates, and ε is the numerical-stability constant used by Adam.

During the training process, the epoch-wise step learning-rate decay strategy is used to control the parameter update amplitude, consistent with the setting that the learning rate decays to 0.95 times its previous value every 10 epochs:


$$\begin{array}{c} \eta_{e}=\eta_{\text{0}}\gamma^{n_{e}} \end{array}$$ (16)

In Equation (16), $\eta_{e}$ denotes the learning rate at epoch e, $\eta_{\text{0}}$ denotes the initial learning rate, γ is the decay coefficient, and $n_{e}$ is the number of completed 10-epoch decay intervals. The experiments use γ = 0.95.

During the training process, the input features extracted through data augmentation and multi-scale attention modules are simultaneously subjected to detection classification loss, bounding box regression loss, RPN candidate region loss, and PAL constraints. Joint optimization makes the features close to the category prototype in high-dimensional space and ensures consistency among the samples.

Table 5 summarizes the optimizer, learning rate, batch size, joint loss weights, training budget, regularization, learning-rate decay, Adam parameters, and data-augmentation settings used in the few-shot experiments.

**Table 5:** PA-MSAN training hyperparameters under the few-shot protocol.

Parameter Category	Parameter	Value/ Setting	Description
Optimizer	Optimizer	Adam	Adaptive first- and second-moment estimation
Learning Rate	Initial Learning Rate	0.001	Initial learning rate with epoch-wise step decay
Batch Size	Batch Size	16	Training views per optimizer step; the K-shot count refers to distinct annotated support images
Loss Function	Joint Loss Weights	Detection: 1.0/ Regression: 1.0/ RPN: 0.5/ Prototype Alignment: 0.5	Weights of detection classification, bounding-box regression, region proposal, and prototype alignment losses
Training Epochs	Epochs	200	200-epoch training budget; best-validation checkpoint used for evaluation
Regularization	Dropout	0.3	Dropout regularization during training
Learning Rate Strategy	Learning Rate Decay	0.95 every 10 epochs	Multiplies the learning rate by 0.95 after each 10-epoch interval
Adam Parameters	First Moment Decay, Second Moment Decay, Epsilon	0.9, 0.999, 1 × 10 ^− 8^	Controls momentum update and numerical stability
Data Augmentation	Augmentation	Rotation ±30°; horizontal/vertical flip; scaling 0.8–1.2; illumination gain 0.8–1.2 and offset −10–10; Gaussian noise std. 0.0–0.05 after normalization	Applied to support-image training views; validation and test preprocessing is deterministic

During training, Adam uses an initial learning rate of 0.001 with a decay factor of 0.95 every 10 epochs. Each optimizer step processes a mini-batch of 16 training views derived from the K-shot support images. The K-shot count refers to distinct annotated source images; augmented views do not increase the shot count. The joint loss weights for detection classification, bounding-box regression, RPN proposal loss, and prototype alignment are 1.0, 1.0, 0.5, and 0.5, respectively, and Dropout is set to 0.3. The prototype-alignment weight $\lambda_{p}$ is selected by validation-set grid search over {0.1, 0.3, 0.5, 0.8, 1.0}, with the remaining weights fixed as in Table 5. Section 4.3.3 reports the corresponding sensitivity results.

## 3. Experimental design and data preparation

### 3.1. Experimental datasets and acquisition

This experiment used two publicly available industrial surface defect detection datasets, NEU-DET [22,23] and GC10-DET [27], to train and evaluate the proposed model. NEU-DET is a steel surface defect detection dataset released by Northeastern University and contains six typical defect categories, including Crazing, Scratch, Pitted Surface, Patch, Rolled-in Scale, and Inclusion, with bounding-box annotations. GC10-DET contains ten defect categories (punching, weld line, crescent gap, water spot, oil spot, silk spot, inclusion, rolled pit, crease, and waist folding) with bounding-box annotations and is used to evaluate cross-domain adaptation with limited target-domain supervision in more complex industrial scenarios.

NEU-DET images are grayscale steel surface images with a resolution of 200 × 200, whereas GC10-DET contains grayscale metallic surface-defect images with variable resolutions in commonly used releases. For a consistent input setting, all images from both datasets are read or converted into a single-channel grayscale format, resized to 416 × 416, and normalized to the 0–1 interval. Random augmentation, including rotation, horizontal/vertical flipping, scaling, illumination gain and offset, and Gaussian noise injection, is applied only to training and support-set images; validation and test images undergo deterministic resizing and normalization only.

Each dataset is divided once into 70% training, 15% validation, and 15% test subsets. For a given run, the 70% training subset is the candidate pool from which exactly K annotated support images per class are sampled. Parameter updates use only these support images and their augmented views. The validation and test subsets remain held out from parameter learning.


$$\begin{array}{c} p_{i}=\frac{N_{i}}{N_{\text{total}}} \end{array}$$ (17)

In Equation (17), $p_{i}$ denotes the proportion of samples from class i, $N_{i}$ denotes the number of samples in that class, and $N_{total}$ is the total sample count. This quantity is used to check category balance before K-shot support sampling.

Image normalization and bounding-box transformations are applied consistently with the geometric augmentation operations so that defect annotations remain aligned with the transformed images. The resulting inputs are then used by the multi-scale feature extractor, attention modules, prototype branch, and detection head.

Table 6 presents the statistical information of the NEU-DET and GC10-DET datasets, including the number of defect categories, annotation forms, image types and application scenarios.

**Table 6:** Statistics of industrial surface defect detection dataset.

Dataset	Defect Categories	Annotation Type	Original Image Type	Original Resolution	Preprocessing in This Study
NEU-DET	6 classes: crazing, scratch, pitted surface, patch, rolled-in scale, inclusion	Bounding box	Grayscale	200 × 200	Resized to 416 × 416; normalized to [0,1]; used as single-channel input
GC10-DET	10 classes: punching, weld line, crescent gap, water spot, oil spot, silk spot, inclusion, rolled pit, crease, waist folding	Bounding box	Grayscale metallic surface-defect images	Variable resolutions	Read/converted as single-channel grayscale; resized to 416 × 416; normalized to 0–1; used for cross-domain evaluation

Table 6 shows the datasets used in this experiment, as well as the number of categories, annotation types, original image formats, original resolutions and preprocessing strategies. NEU-DET is used mainly for steel surface defect detection and few-shot evaluation, while GC10-DET is used to evaluate cross-domain adaptation with limited target-domain supervision under more complex industrial imaging conditions.

#### 3.1.1. Few-shot episode construction and evaluation protocol.

For each defect category, K annotated images are randomly sampled from the fixed 70% training subset as the support set, with K ∈ {1, 3, 5}. These K source images and their stochastic augmented views provide the labeled training material for that run. The dedicated 15% validation and 15% test subsets remain non-overlapping with the support pool and are used only for model selection and final evaluation, respectively.

For the cross-domain experiment reported in Fig 10, five labeled GC10-DET support images per class are selected from the target-domain training pool. PA-MSAN constructs target-domain class prototypes from these 5-shot support samples, and performance is evaluated on the fixed non-overlapping GC10-DET test subset.

Each shot setting is repeated five times with random seeds {0, 1, 2, 3, 4}. PA-MSAN and all baselines use the same seed-specific support selections, preprocessing pipeline, confidence threshold, IoU threshold, and non-maximum suppression (NMS) threshold. Across seeds, support sampling, augmentation draws, mini-batch order, and Dropout masks are re-randomized while the underlying 70%/15%/15% split remains fixed. Each repeated run starts from the architecture-specific initialization or pretrained state listed in Table 7. Results are summarized as mean ± standard deviation over the five runs.

**Table 7:** Baseline implementation details under the shared evaluation protocol.

Model	Input size	Initialization/ Pretraining	Data and augmentation	Training protocol	Thresholds	Evaluation protocol
CNN Detector	416 × 416	Xavier initialization	Same split and augmentation	Adam, 200 epochs, batch size 16	IoU/NMS/conf.: 0.5/0.5/0.25	Same seeds 0–4; mean ± std
Proto CNN	416 × 416	Xavier initialization	Same split and augmentation	Adam, 200 epochs, batch size 16	IoU/NMS/conf.: 0.5/0.5/0.25	Same seeds 0–4; mean ± std
Attention CNN	416 × 416	Xavier initialization	Same split and augmentation	Adam, 200 epochs, batch size 16	IoU/NMS/conf.: 0.5/0.5/0.25	Same seeds 0–4; mean ± std
YOLOv5	416 × 416	COCO-pretrained weights	Same data split and augmentation	Official training protocol; 200 epochs	IoU/NMS/conf.: 0.5/0.5/0.25	Same seeds 0–4; mean ± std
Faster R-CNN	416 × 416	ImageNet-pretrained ResNet50 backbone	Same data split and augmentation	Official training protocol; 200 epochs	IoU/NMS/conf.: 0.5/0.5/0.25	Same seeds 0–4; mean ± std
TFA [19]	416 × 416	Base-detector pretrained checkpoint	Same split and augmentation	Two-stage fine-tuning per official protocol	IoU/NMS/conf.: 0.5/0.5/0.25	Same seeds 0–4; mean ± std
Meta R-CNN [21]	416 × 416	ImageNet-pretrained backbone	Same split and augmentation	Official implementation; fine-tuned on K-shot support	IoU/NMS/conf.: 0.5/0.5/0.25	Same seeds 0–4; mean ± std
FSRW [20]	416 × 416	ImageNet-pretrained backbone	Same split and augmentation	Official implementation; feature-reweighting fine-tuning	IoU/NMS/conf.: 0.5/0.5/0.25	Same seeds 0–4; mean ± std
PA-MSAN	416 × 416	Xavier initialization	Same split and augmentation	Adam, 200 epochs, batch size 16	IoU/NMS/conf.: 0.5/0.5/0.25	Same seeds 0–4; mean ± std

For example, 92.2 ± 0.4% mAP@0.5 in the 5-shot setting denotes the mean and standard deviation across the five seed-specific runs. This reporting separates average performance from variability caused by support selection and other stochastic training factors.

Table 8 summarizes the 1-shot, 3-shot, and 5-shot episode settings. Each configuration uses balanced support counts and identical seed-specific support selections across compared methods, allowing performance changes with increasing support size to be evaluated under the same data partition and detection thresholds.

**Table 8:** Few-shot episode construction and evaluation protocol.

Shot setting	Support samples per class	Random seeds	Repeated runs	Held-out evaluation	Reported metric
1-shot	1 annotated image per defect class	0, 1, 2, 3, 4	5	Validation: checkpoint; Test: final	mean ± std
3-shot	3 annotated images per defect class	0, 1, 2, 3, 4	5	Validation: checkpoint; Test: final	mean ± std
5-shot	5 annotated images per defect class	0, 1, 2, 3, 4	5	Validation: checkpoint; Test: final	mean ± std

Note: Support images are non-overlapping with the held-out validation and test images in each run. Validation images are used for checkpoint selection and test images for final evaluation. Results are reported as mean ± standard deviation over seeds 0–4.

### 3.2. Experimental configuration and training settings

Experiments are conducted on Ubuntu 20.04 using an NVIDIA GeForce RTX 3090 graphics processing unit (GPU) with 24 GB memory and an Intel Xeon Gold 6338 central processing unit (CPU). CUDA acceleration is used for training and inference. All images are resized to 416 × 416 and normalized to the 0–1 interval before entering the network.

Inference-time measurements use batch size 1 with 416 × 416 inputs on the RTX 3090. Detection time includes network forward inference and detection post-processing/NMS and excludes disk input/output (I/O) and input preprocessing.

PA-MSAN consists of a multi-scale convolutional backbone with feature-pyramid fusion, spatial and channel attention, an RoI-level prototype branch, RPN proposal generation, and a detection head. The attention-enhanced pyramid representation is used by the RPN and RoI Align stages. The resulting RoI features are processed by the prototype metric head for few-shot class prediction and by the box-regression branch for localization; the auxiliary linear classification head provides the Ldet supervision during training.

Training uses mini-batch stochastic optimization with batch size 16. Adam is initialized with a learning rate of 1 × 10^−3^, which is multiplied by 0.95 every 10 epochs. The optimizer uses adaptive first- and second-moment estimates as specified in Table 5.

During inference, the prototype class probability from Equation (11) is combined with the RPN objectness probability to form the final detection confidence:


$$\begin{array}{c} s_{\text{conf}}=P\left(\text{obj}\right)\cdot P\left(\text{cls}|\text{obj}\right) \end{array}$$ (18)

In Equation (18), $s_{\text{conf}}$ denotes the final detection confidence score, P(obj) is the candidate-region objectness probability, and P(cls∣obj) is the prototype-based conditional class probability. During NMS, IoU is computed between predicted boxes; evaluation IoU is computed between matched predicted and ground-truth boxes.

During validation and testing, detections are evaluated at an IoU threshold of 0.5, with an NMS threshold of 0.5 and a confidence threshold of 0.25. Each training run uses a 200-epoch budget, and the checkpoint with the highest validation mAP is used for final test evaluation. A Dropout rate of 0.3 is applied to the fully connected layer during training. Table 9 reports the seed-aggregated epoch at which the best validation mAP is reached for each prototype-alignment weight.

**Table 9:** Sensitivity of PA-MSAN to the Prototype Alignment Loss weight $\lambda_{p}$ on NEU-DET.

$\lambda_{p}$	1-shot mAP@0.5 (%)	3-shot mAP@0.5 (%)	5-shot mAP@0.5 (%)	Epochs to best val. mAP
0.1	83.2 ± 0.6	88.1 ± 0.5	91.0 ± 0.5	172 ± 3.5
0.3	84.1 ± 0.4	88.9 ± 0.4	91.7 ± 0.4	165 ± 3.4
0.5	84.6 ± 0.3	89.3 ± 0.3	92.2 ± 0.4	158 ± 3.1
0.8	84.0 ± 0.5	88.8 ± 0.4	91.6 ± 0.5	183 ± 3.2
1.0	83.5 ± 0.6	88.4 ± 0.5	91.1 ± 0.6	196 ± 3.8

Note: Values are reported as mean ± standard deviation over seeds 0–4. $\lambda_{\text{p}}$ = 0.5 is the setting used in Table 5.

### 3.3. Model comparison and ablation settings

Comparative and ablation experiments use a shared target-dataset partition, input resolution, support-set protocol, augmentation policy, random seeds, and evaluation thresholds. Each baseline retains its prescribed model-specific optimization procedure while using the same target-dataset support selections and evaluation conditions.

The comparison includes three in-house reference variants (CNN Detector, Proto CNN, and Attention CNN), two general-purpose detectors (YOLOv5 [44] and Faster R-CNN [45]), and three established few-shot object detectors (TFA [19], Meta R-CNN [21], and FSRW [20]). CNN Detector uses a conventional CNN-FPN detection pipeline without attention or prototype learning; Proto CNN adds prototype constraints; and Attention CNN adds spatial-channel attention without the prototype branch. TFA, Meta R-CNN, and FSRW provide few-shot-specific references with classifier fine-tuning, RoI-level meta features, and feature reweighting, respectively. PA-MSAN is evaluated under the same episode and detection-evaluation protocol as these baselines.

All models use the same dataset split, 5-shot GC10-DET support selections, 416 × 416 input resolution, confidence threshold of 0.25, NMS threshold of 0.5, and random seeds. CNN Detector, Proto CNN, Attention CNN, and PA-MSAN use Adam for 200 epochs with an initial learning rate of 1 × 10^−3^ and batch size 16. YOLOv5, Faster R-CNN, TFA, Meta R-CNN, and FSRW follow their method-specific training procedures within the same 200-epoch budget. For cross-domain evaluation, PA-MSAN constructs target-domain prototypes from the support set, while each baseline uses the same labeled support set according to its method-specific adaptation procedure.

For target-domain evaluation, every compared method uses the same five labeled GC10-DET support images per class for its method-specific adaptation stage and is evaluated on the same fixed non-overlapping GC10-DET test subset.

#### 3.3.1. Baseline implementation details.

Table 7 summarizes the implementation and evaluation settings used for the compared detectors. Each repeated run starts independently from the initialization or pretraining configuration listed in Table 7.

The shared split, support episodes, preprocessing, thresholds, and random seeds align the target-dataset evaluation conditions across methods. Reported differences are considered together with each detector’s architecture and prescribed optimization procedure.

The ablation study removes the spatial attention module, channel attention module, prototype-learning branch, Prototype Alignment Loss, and multi-scale feature extraction structure in controlled variants. All variants use the same data split, random seeds, detection head, training budget, optimizer settings, augmentation policy, and evaluation thresholds. Table 10 summarizes the corresponding configurations.

**Table 10:** Comparison methods and ablation experimental setup.

Experiment Type	Backbone Structure	Attention Module	Prototype Learning	Detection Head	Training Strategy
CNN Detector	Standard CNN + FPN	None	None	Classification + Box Regression	Adam optimizer, batch size 16
Proto CNN	CNN + FPN	None	Enabled	Classification + Box Regression	Adam optimizer, batch size 16
Attention CNN	CNN + FPN	Spatial + Channel Attention	None	Classification + Box Regression	Adam optimizer, batch size 16, learning rate 1 × 10 − 3
YOLOv5 Baseline	CSPDarknet + Path Aggregation Network (PAN)/FPN aggregation	No explicit attention	None	YOLO Detection Head	Method-specific training; shared evaluation protocol
Faster R-CNN	ResNet50-FPN	None	None	Region Proposal + Regression	Method-specific training; shared evaluation protocol
Ablation – w/o spatial attention (SA)	Full Network	Without Spatial Attention	Enabled	Unified Detection Head	Other parameters unchanged
Ablation – w/o channel attention (CA)	Full Network	Without Channel Attention	Enabled	Unified Detection Head	Other parameters unchanged
Ablation – w/o prototype learning (PL)	Full Network	Spatial + Channel Attention	Disabled	Unified Detection Head	Other parameters unchanged
Ablation – w/o MSFE	Single-scale CNN	Spatial + Channel Attention	Enabled	Unified Detection Head	Other parameters unchanged
Ablation – w/o PAL	Full Network	Spatial + Channel Attention	Enabled (prototype classifier retained)	Unified Detection Head	PAL loss removed; other parameters unchanged
Full Model	Multi-scale CNN + FPN	Spatial + Channel Attention	Enabled	Unified Detection Head	Complete proposed framework

Detection performance is evaluated with average precision (AP) and mAP@0.5, with mAP@0.5 used as the primary metric for combined localization and classification performance. For an ablation variant, the mAP decrease relative to the complete model is defined as:


$$\begin{array}{c} \Delta \text{maP}={\text{mAP}}_{\text{full}}-{\text{mAP}}_{\text{ablated}} \end{array}$$ (19)

In Equation (19), ΔmAP denotes the performance decrease associated with removing the corresponding component from the full model, ${\text{mAP}}_{\text{full}}$ is the full-model performance, and ${\text{mAP}}_{\text{ablated}}$ is the performance of the ablated variant.

## 4. Result analysis

### 4.1. Detection performance under multi-scale and attention mechanisms

#### 4.1.1. Multi-scale defect detection performance evaluation.

PA-MSAN is evaluated on six NEU-DET defect classes using average precision at an IoU threshold of 0.5 (AP50), scale-specific AP, and mAP@0.5. Object-scale groups are defined after resizing to the 416 × 416 network input. If A denotes the resized ground-truth bounding-box area in pixels, small objects satisfy $A<{\text{3}\text{2}}^{\text{2}}$, medium objects satisfy ${\text{3}\text{2}}^{\text{2}}\leq A<{\text{6}\text{4}}^{\text{2}}$, and large objects satisfy $A\geq {\text{6}\text{4}}^{\text{2}}$. mAP@0.5 is the mean AP50 over the six classes, and ${\text{AP}}_{\text{small}}$ denotes AP at IoU = 0.5 evaluated only on the small-object group.


$$\begin{array}{c} \text{AP}=\int_{\text{0}}^{\text{1}}P\left(R\right)\;dR,\;{\text{mAP}}_{\text{0}.\text{5}}=\frac{\text{1}}{K}\sum_{k=\text{1}}^{K}{\text{AP}}_{k,\text{IoU}=\text{0}.\text{5}} \end{array}$$ (20)

In Equation (20), *P(R)* denotes precision as a function of recall R, *AP* is the area under the precision-recall curve, *K* denotes the number of defect categories, and ${\text{mAP}}_{\text{0}.\text{5}}$ denotes the mean AP across categories when the IoU threshold is 0.5. Representative multi-scale responses are shown in Fig 6, and category-wise AP50 results are summarized in Fig 7.

**Fig 6:**
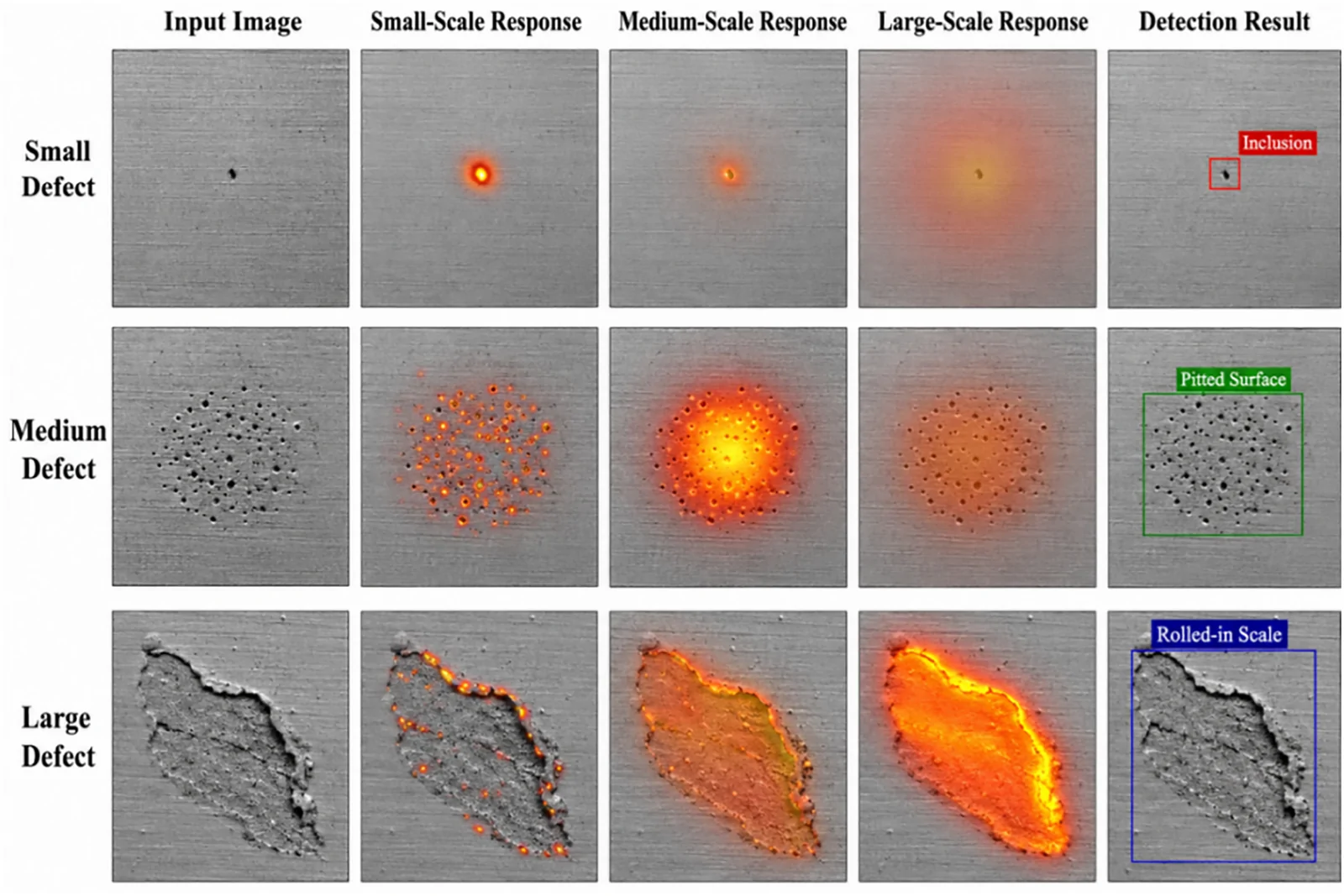
Detection results of small-, medium-, and large-scale defects in the multi-scale detection structure. Source material, where applicable: Reprinted from the NEU-DET dataset [22,23] under a CC BY 4.0 license, with permission from Kechen Song, original copyright 2013. Feature visualizations, annotations, and figure composition were generated by the authors.

**Fig 7:**
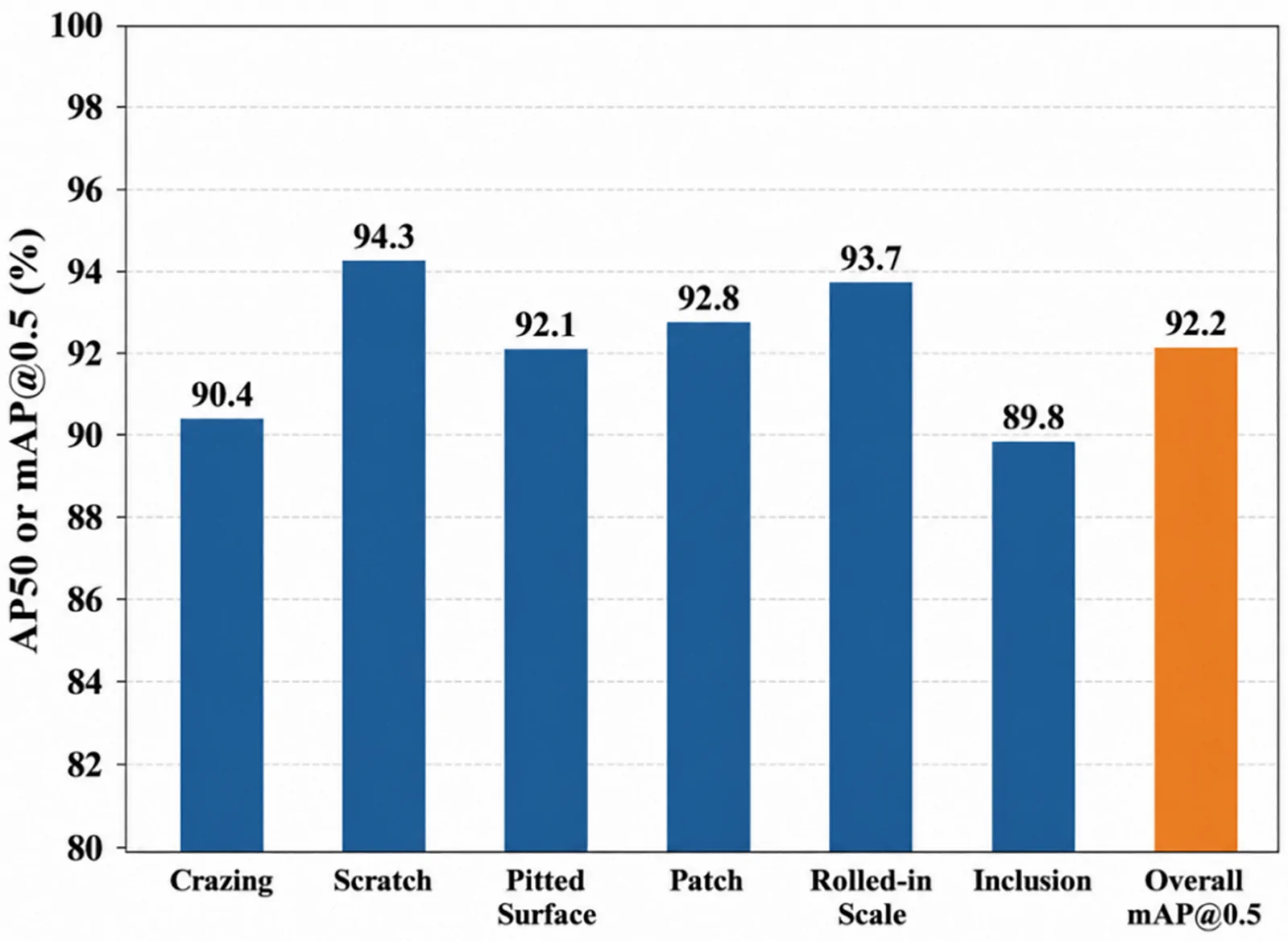
Category-wise 5-shot AP50 and overall mAP@0.5 of PA-MSAN on NEU-DET.

Fig 6 illustrates complementary responses of the MSFE module across defect scales. Inclusion retains a concentrated response in the fine-scale representation, Pitted Surface is represented more continuously at the intermediate scale, and Rolled-in Scale receives a broader response from the large-receptive-field branch. The examples are consistent with the intended role of the multi-scale pathway: preserving local texture details while incorporating larger spatial context for extended defects.

Fig 7 reports the mean category-wise AP50 values over five repeated few-shot runs together with the overall mAP@0.5 of 92.2%. Scratch and Rolled-in Scale reach 94.3% and 93.7% AP50, respectively, while Crazing and Inclusion show lower category-wise AP50 under the same evaluation protocol.

#### 4.1.2. Attention focus detection effect.

The contribution of SA and CA is evaluated using no-attention, SA-only, CA-only, and dual-attention variants on the six NEU-DET classes. Localization quality is measured from matched-box IoU. Fig 8 also reports boundary integrity, background false response rate, and target/non-target channel-response intensity as implementation-level diagnostic quantities.

**Fig 8:**
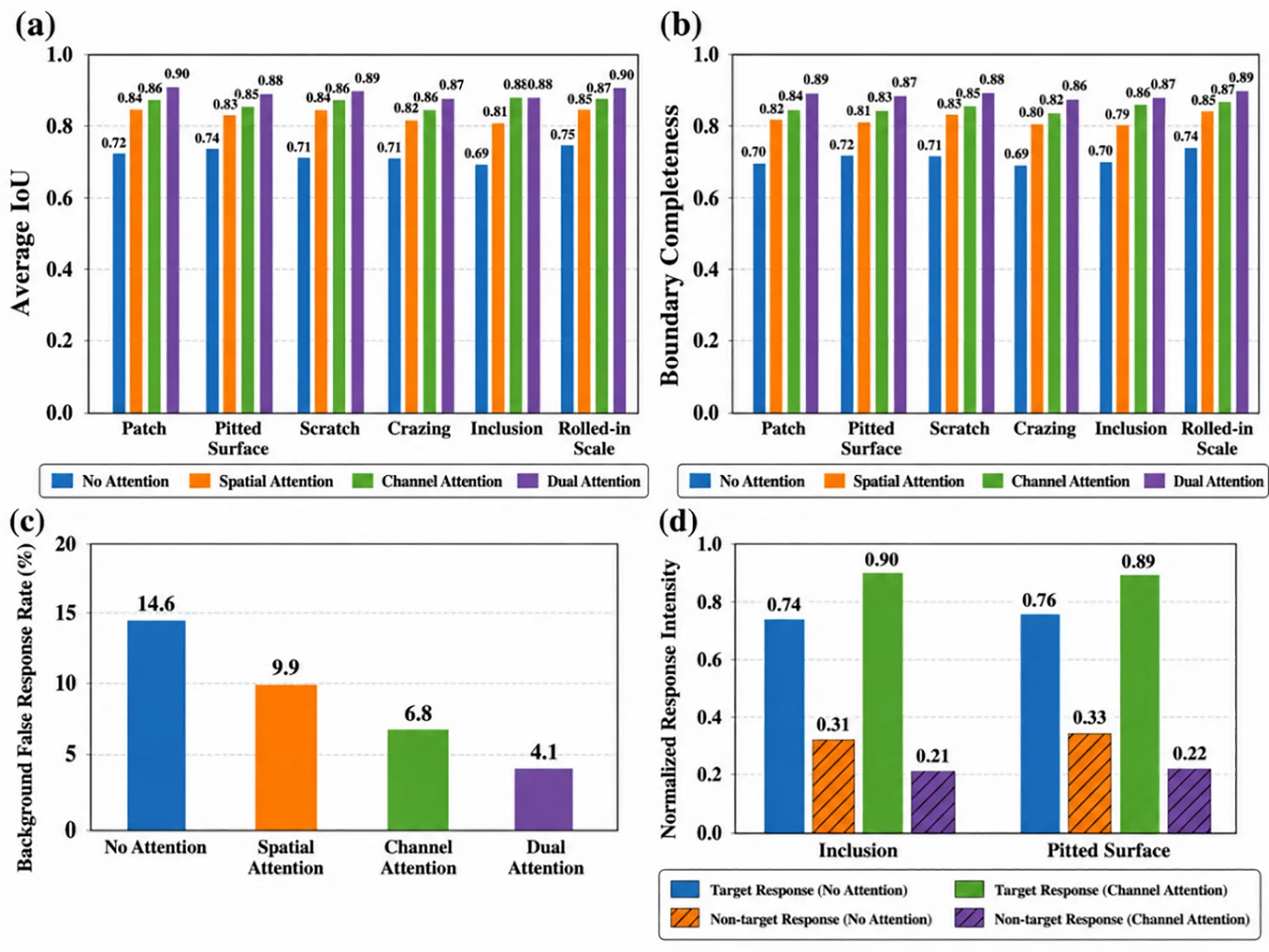
Quantitative comparison of attention-enhancement effects on localization quality, boundary integrity score, background suppression, and channel-response separation. (a) Category-wise Average IoU under different attention settings. (b) Category-wise boundary integrity score under different attention settings. (c) Overall background false response rate under different attention settings. (d) Target and non-target response intensity before and after channel attention.

The calculation method of IoU is as follows:


$$\begin{array}{c} \text{IoU}=\frac{\left|B_{\text{pred}}\cap B_{\text{gt}}\right|}{\left|B_{\text{pred}}\cup B_{\text{gt}}\right|} \end{array}$$ (21)

In Equation (21), $B_{\text{pred}}$ denotes the predicted bounding box and $B_{\text{gt}}$ denotes the ground-truth bounding box. A larger IoU indicates more accurate target localization. The quantitative results are shown in Fig 8.

For localization-quality reporting, predictions are sorted by confidence and greedily matched to ground-truth boxes at IoU ≥ 0.5, with each ground-truth instance matched at most once. Each ground-truth instance contributes the IoU of its matched prediction, or zero if it is missed. Extra unmatched predictions are counted as false positives for precision/AP computation but are not added as separate terms to the Average-IoU denominator. For images with multiple objects, these ground-truth-level IoU contributions are pooled across the full test set and averaged as in Equation (22).


$$\begin{array}{c} \overset{―}{\text{IoU}}=\frac{\text{1}}{N_{m}}\sum_{i=\text{1}}^{N_{m}}\text{IoU}\left(B_{p,i},B_{g,i}\right) \end{array}$$ (22)

In Equation (22), $N_{m}$ denotes the total number of ground-truth instances included in the Average-IoU calculation. For each instance i, $B_{p,i}$ is the matched prediction when one exists and $B_{g,i}$ is the ground-truth box; missed instances contribute an IoU value of zero. IoU(·,·) follows Equation (21).

Fig 8(a) compares category-wise Average IoU across the four attention settings. Fig 8(b) reports boundary integrity, Fig 8(c) reports the overall background false response rate, and Fig 8(d) compares target and non-target channel-response intensity. Boundary integrity is reported as a boundary-agreement score; background false response rate records background detections above the evaluation confidence threshold; and target/non-target response intensity summarizes normalized activation in target and non-target regions. Across these diagnostics, the dual-attention configuration shows higher localization-related scores and a lower background false response rate than the no-attention reference.

Boundary integrity score is used as an auxiliary localization diagnostic. Background false response rate is calculated as the number of retained background false-positive detections divided by the total number of retained detections after confidence filtering and NMS. A retained prediction is counted as a background false positive when it cannot be matched to any ground-truth defect at IoU ≥ 0.5.

### 4.2. Few-shot and cross-domain adaptation performance

#### 4.2.1. Few-shot detection performance.

Few-shot performance is evaluated on NEU-DET under the 1-shot, 3-shot, and 5-shot settings defined in Table 8. During test evaluation, RoI features are classified by cosine similarity to prototypes constructed from the corresponding support set. PA-MSAN and all baselines use the same seed-specific support selections and evaluation thresholds. The corresponding few-shot performance and prototype-discriminability results are summarized in Fig 9.

**Fig 9:**
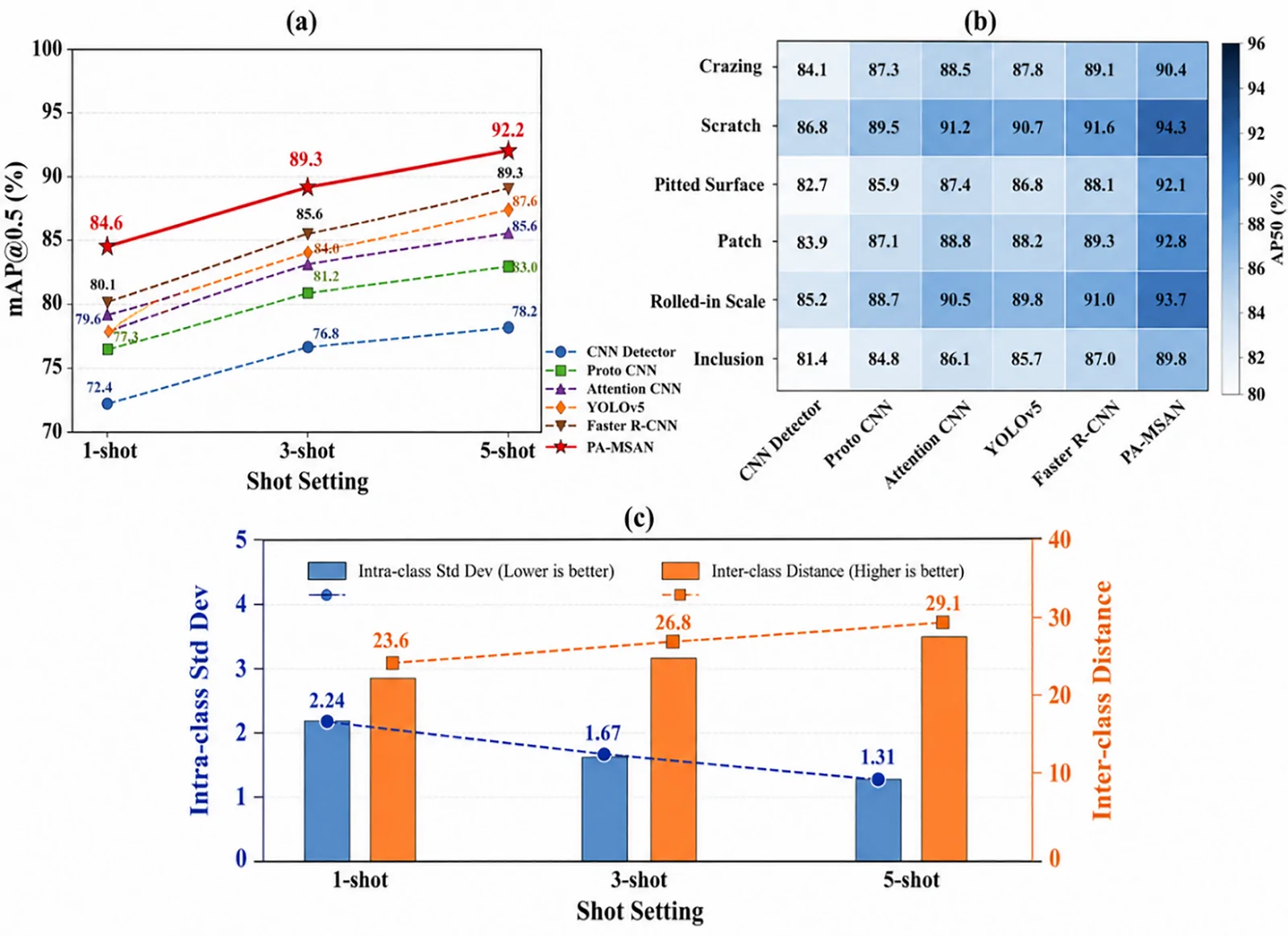
Few-shot detection performance and prototype discriminability under different shot settings. (a) mAP@0.5 trends of different methods under 1-shot, 3-shot, and 5-shot settings. (b) AP50 comparison of different defect categories under the 5-shot setting. (c) Intra-class standard deviation and inter-class distance under different shot settings.

Five seed-level runs are summarized as mean ± standard deviation in the result tables; the corresponding seed-level values are provided in S1 File.

Fig 9(a) reports mean mAP@0.5 under the three shot settings. PA-MSAN reaches 84.6 ± 0.3%, 89.3 ± 0.3%, and 92.2 ± 0.4% mAP@0.5 for 1-shot, 3-shot, and 5-shot, respectively. Fig 9(b) presents category-wise 5-shot AP50, and Fig 9(c) shows lower intra-class standard deviation and larger inter-class distance as the number of support samples increases. These measurements are consistent with more stable class prototypes when additional support examples are available. The detailed quantitative results of PA-MSAN under different few-shot settings are summarized in Table 11.

**Table 11:** Few-shot detection performance of PA-MSAN on NEU-DET.

Shot setting	mAP@0.5 (%)	APsmall (%)	Average IoU	Background false response rate (%)
1-shot	84.6 ± 0.3	79.4 ± 0.5	0.82 ± 0.01	7.8 ± 0.3
3-shot	89.3 ± 0.3	84.6 ± 0.4	0.86 ± 0.01	5.6 ± 0.2
5-shot	92.2 ± 0.4	88.7 ± 0.4	0.89 ± 0.01	4.1 ± 0.2

Note: Values are reported as mean ± standard deviation over five repeated runs using seeds 0–4. Background false response rate is computed from retained post-NMS detections using the matching rule defined in Section 4.1.2.

The comparative results between PA-MSAN and baseline detectors under the 5-shot NEU-DET setting are summarized in Table 12.

**Table 12:** Comparison with baseline methods under the 5-shot NEU-DET setting.

Model	mAP@0.5 (%)	APsmall (%)	Average IoU	Background false response rate (%)
CNN Detector	78.2 ± 0.3	72.4 ± 0.6	0.77 ± 0.01	12.3 ± 0.5
Proto CNN	83.0 ± 0.4	78.0 ± 0.5	0.81 ± 0.01	9.5 ± 0.4
Attention CNN	85.6 ± 0.4	81.5 ± 0.5	0.83 ± 0.01	7.6 ± 0.3
YOLOv5	87.6 ± 0.3	82.9 ± 0.4	0.85 ± 0.01	6.9 ± 0.3
Faster R-CNN	89.3 ± 0.4	84.8 ± 0.4	0.86 ± 0.01	5.8 ± 0.3
TFA [19]	88.4 ± 0.4	84.0 ± 0.5	0.86 ± 0.01	5.9 ± 0.3
Meta R-CNN [21]	86.8 ± 0.5	81.6 ± 0.6	0.83 ± 0.01	7.9 ± 0.4
FSRW [20]	85.9 ± 0.7	80.2 ± 0.7	0.82 ± 0.01	8.8 ± 0.5
PA-MSAN	92.2 ± 0.4	88.7 ± 0.4	0.89 ± 0.01	4.1 ± 0.2

Note: Lower background false response rate is better. All methods are evaluated under the same 5-shot protocol, and values are reported as mean ± standard deviation over five repeated runs using seeds 0–4. Background false response rate is computed from retained post-NMS detections using the matching rule defined in Section 4.1.2.

Table 12 also includes three few-shot-specific detectors. TFA [19], Meta R-CNN [21], and FSRW [20] reach 88.4 ± 0.4%, 86.8 ± 0.5%, and 85.9 ± 0.7% mAP@0.5, respectively, compared with 92.2 ± 0.4% for PA-MSAN. PA-MSAN also records the highest APsmall in this comparison, exceeding TFA by 4.7 percentage points under the shared 5-shot evaluation protocol.

#### 4.2.2. Cross-domain adaptation with limited target-domain supervision.

Cross-domain evaluation uses NEU-DET as the source-domain training dataset and GC10-DET as the target dataset. The reported experiment uses five labeled GC10-DET support images per class for target-domain adaptation and evaluates each method on the fixed non-overlapping GC10-DET test subset. Fig 10 reports the 5-shot target-domain mAP@0.5 together with the corresponding cross-dataset differences relative to the 5-shot NEU-DET results.

**Fig 10:**
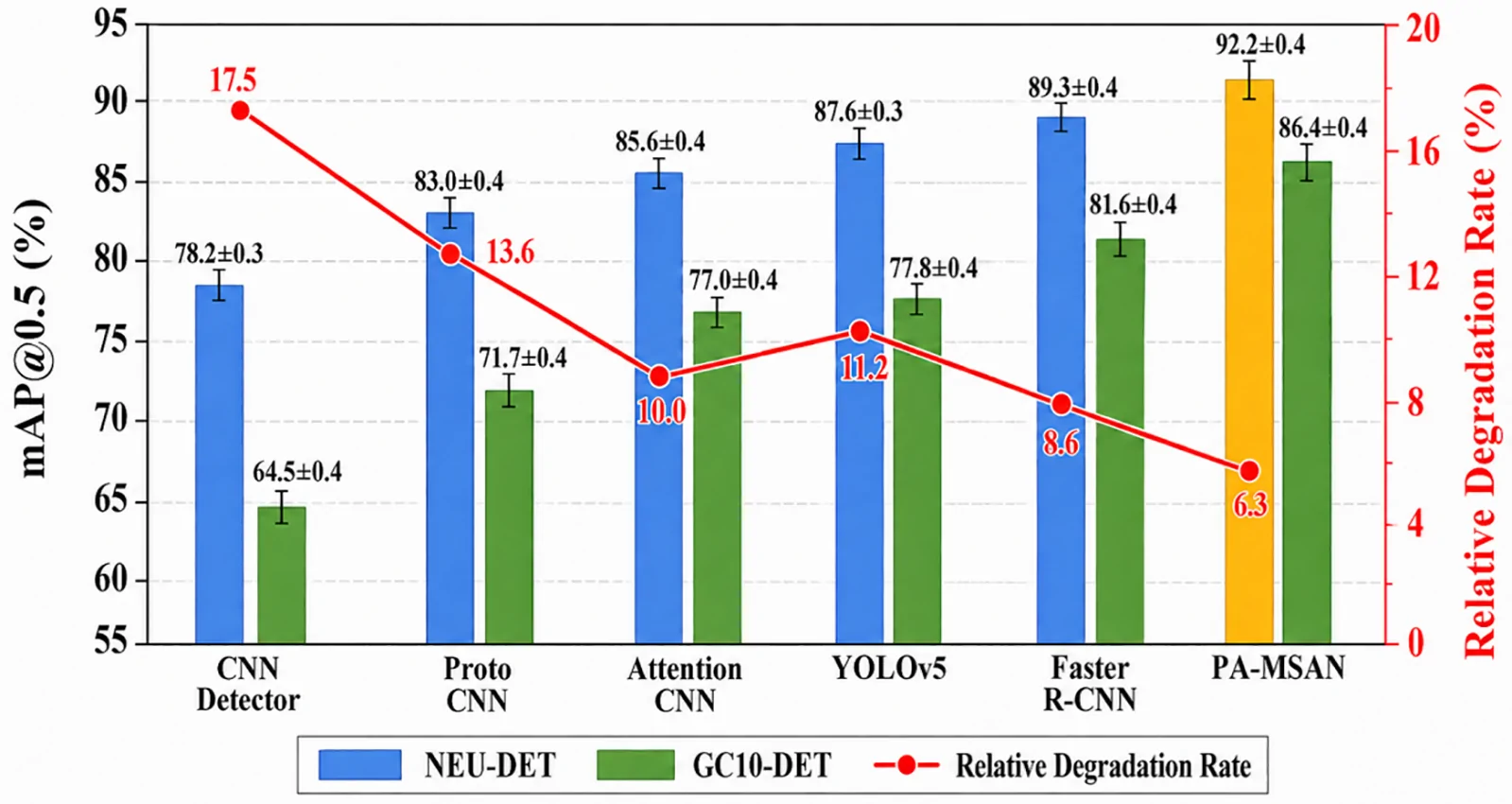
5-shot cross-domain adaptation performance and relative-difference comparison between NEU-DET and GC10-DET.

Under the 5-shot target-domain support setting, PA-MSAN reaches 86.4 ± 0.4% mAP@0.5 on GC10-DET, compared with 92.2 ± 0.4% on NEU-DET. The corresponding difference is 5.8 percentage points (6.3%). The relative differences for Faster R-CNN, Attention CNN, YOLOv5, Proto CNN, and CNN Detector are 8.6%, 10.0%, 11.2%, 13.6%, and 17.5%, respectively. The experiment evaluates adaptation under a change in dataset, target-class composition, image distribution, and object scale using limited labeled target-domain support.

Fig 11 presents four representative GC10-DET failure cases: two false positives and two false negatives. The examples show errors associated with defect-like background texture, illumination boundaries, low-contrast linear structure, and fine localized defect patterns.

**Fig 11:**
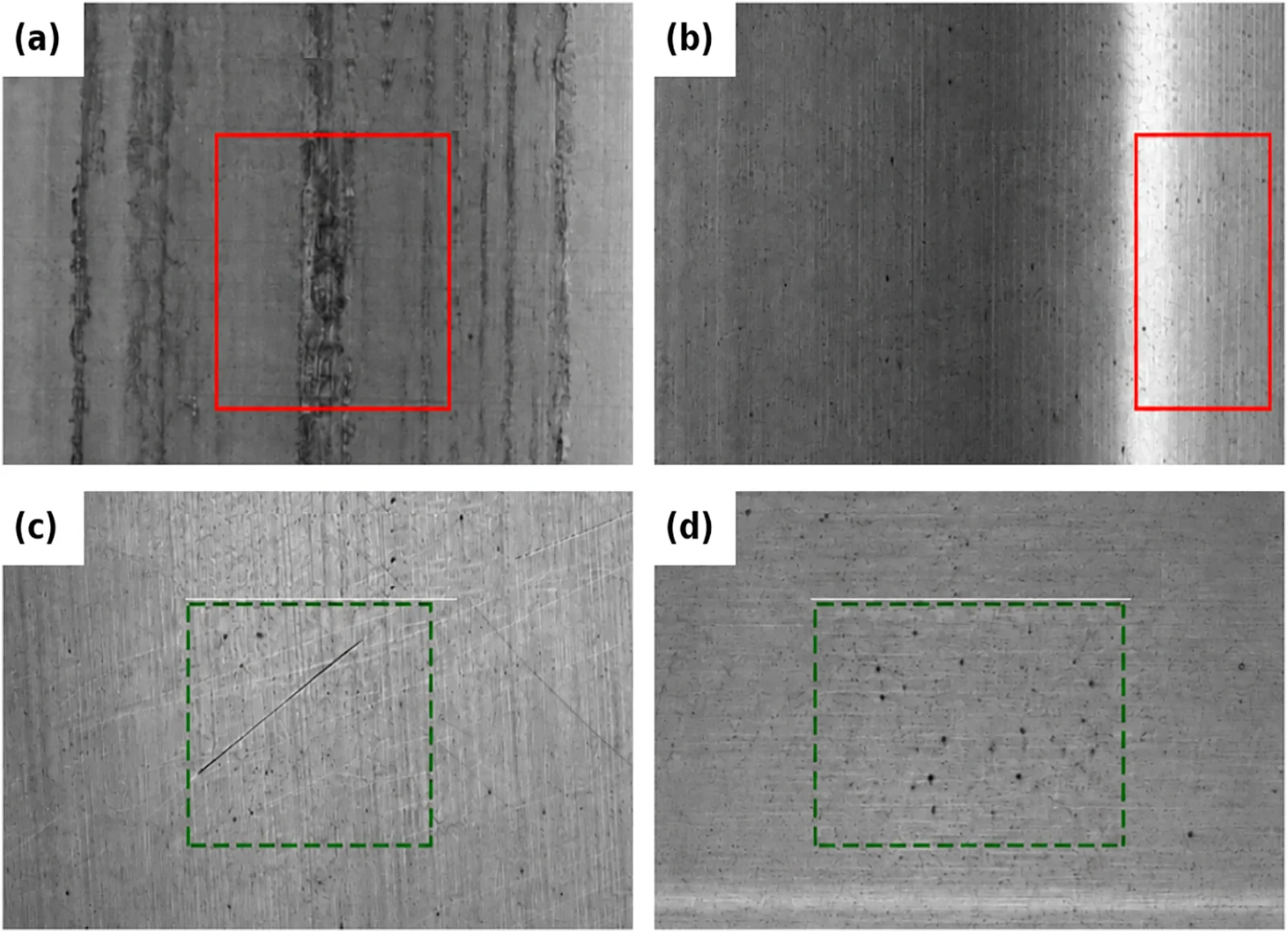
Representative 5-shot cross-domain failure cases of PA-MSAN on GC10-DET. Source material, where applicable: Reprinted from the GC10-DET dataset [27] under a CC BY 4.0 license, with permission from Xiaoming Lv, original copyright 2020. Detection results, annotations, and figure composition were generated by the authors. (a) False positive caused by defect-like background texture. (b) False positive caused by illumination-edge interference. (c) Missed detection of a low-contrast linear defect. (d) Missed detection of a fine localized defect pattern.

Fig 11 shows two recurring error patterns in the target-domain evaluation. False positives occur when periodic textures or illumination boundaries produce RoI features close to defect prototypes. False negatives occur when low-contrast or fine-scale defect regions yield weak region responses and reduced feature-prototype similarity at the detection threshold. These cases motivate adaptive or multi-prototype representations for classes with larger appearance variation.

### 4.3. Small-defect and comprehensive ablation analysis

#### 4.3.1. Small defect detection capability.

Small-defect performance is evaluated on representative NEU-DET categories including Crazing, Pitted Surface, and Inclusion. The comparison uses APsmall, localization error, boundary integrity score, and detection time together with attention-response and box-overlay visualizations. The same scale definition as Section 4.1.1 is used throughout: at the resized 416 × 416 input, small objects satisfy A < 32², medium objects satisfy 32² ≤ A < 64², and large objects satisfy A ≥ 64².

Localization error is computed over matched small-defect instances as the Euclidean distance, in pixels, between the centers of predicted and ground-truth bounding boxes for pairs matched at IoU ≥ 0.5. The reported localization error is the instance-level mean over the evaluated subset. Boundary integrity score is reported as an auxiliary localization diagnostic. Representative qualitative cases are shown in Fig 12.

**Fig 12:**
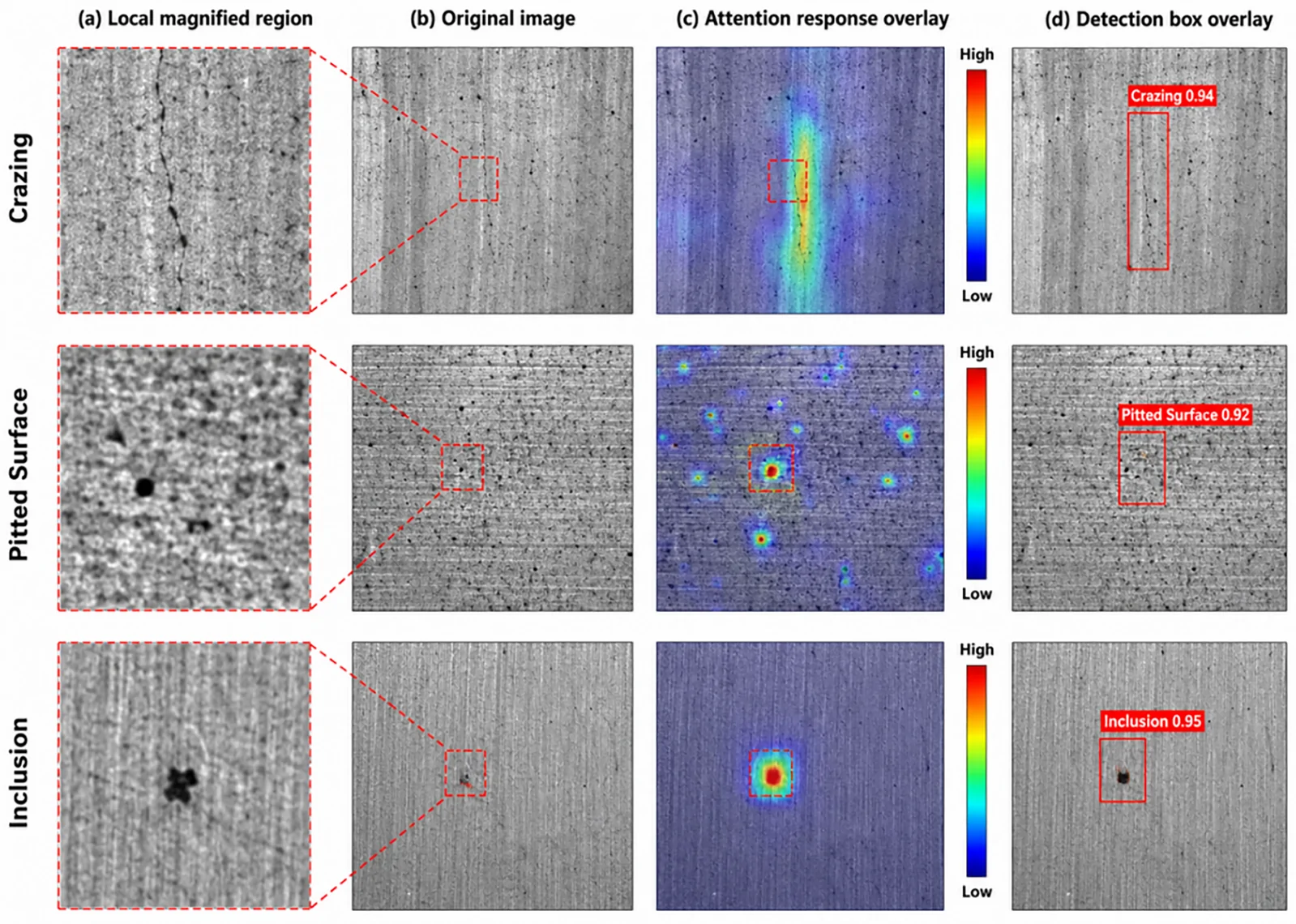
Local magnification and attention-response visualization of representative small defects. Source material, where applicable: Reprinted from the NEU-DET dataset [22,23] under a CC BY 4.0 license, with permission from Kechen Song, original copyright 2013. Feature visualizations, annotations, and figure composition were generated by the authors. (a) Local magnified region. (b) Original image. (c) Attention-response overlay. (d) Detection-box overlay.

Fig 12 shows representative Crazing, Pitted Surface, and Inclusion cases with small or fragmented regions. The attention-response overlays concentrate more strongly around the annotated defect areas, and the detection-box overlays show the corresponding predicted locations. These qualitative examples complement the quantitative small-object results reported in Fig 13.

**Fig 13:**
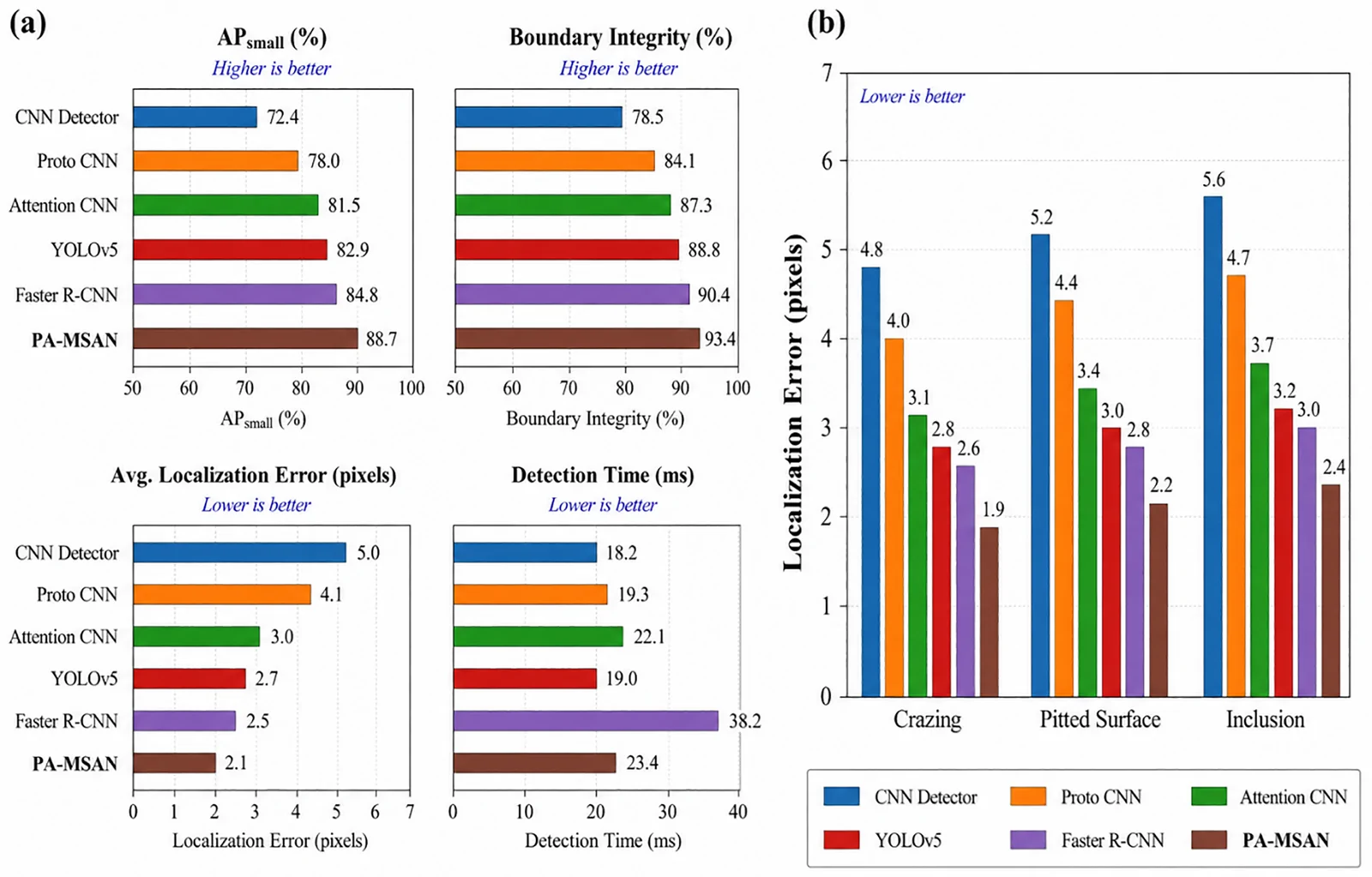
Quantitative comparison of small-defect detection accuracy, localization, boundary integrity score, and detection time. (a) Overall comparison of APsmall, boundary integrity score, localization error, and detection time for small-defect detection. (b) Category-wise localization error comparison for representative small defects.

Fig 13(a) reports 88.7 ± 0.4% APsmall for PA-MSAN, 5.8 percentage points above YOLOv5 and 16.3 percentage points above CNN Detector. PA-MSAN also records a localization error of 2.1 pixels, a boundary integrity score of 93.4%, and a detection time of 23.4 ms. Fig 13(b) reports category-wise localization error for representative small defects. These measurements summarize the accuracy-localization-computation profile of the complete model on the evaluated small-defect subset.

#### 4.3.2. Comprehensive performance and ablation analysis.

Controlled 5-shot ablations quantify the contributions of the multi-scale feature extractor, prototype-learning branch, Prototype Alignment Loss, spatial attention, and channel attention on NEU-DET. All variants use the same data split, seeds, detection head, training budget, optimizer parameters, augmentation policy, confidence threshold, and NMS threshold.

The quantitative ablation results under the 5-shot NEU-DET setting are summarized in Table 1. The corresponding overall performance and controlled-ablation visualization is shown in Fig 14.

**Fig 14:**
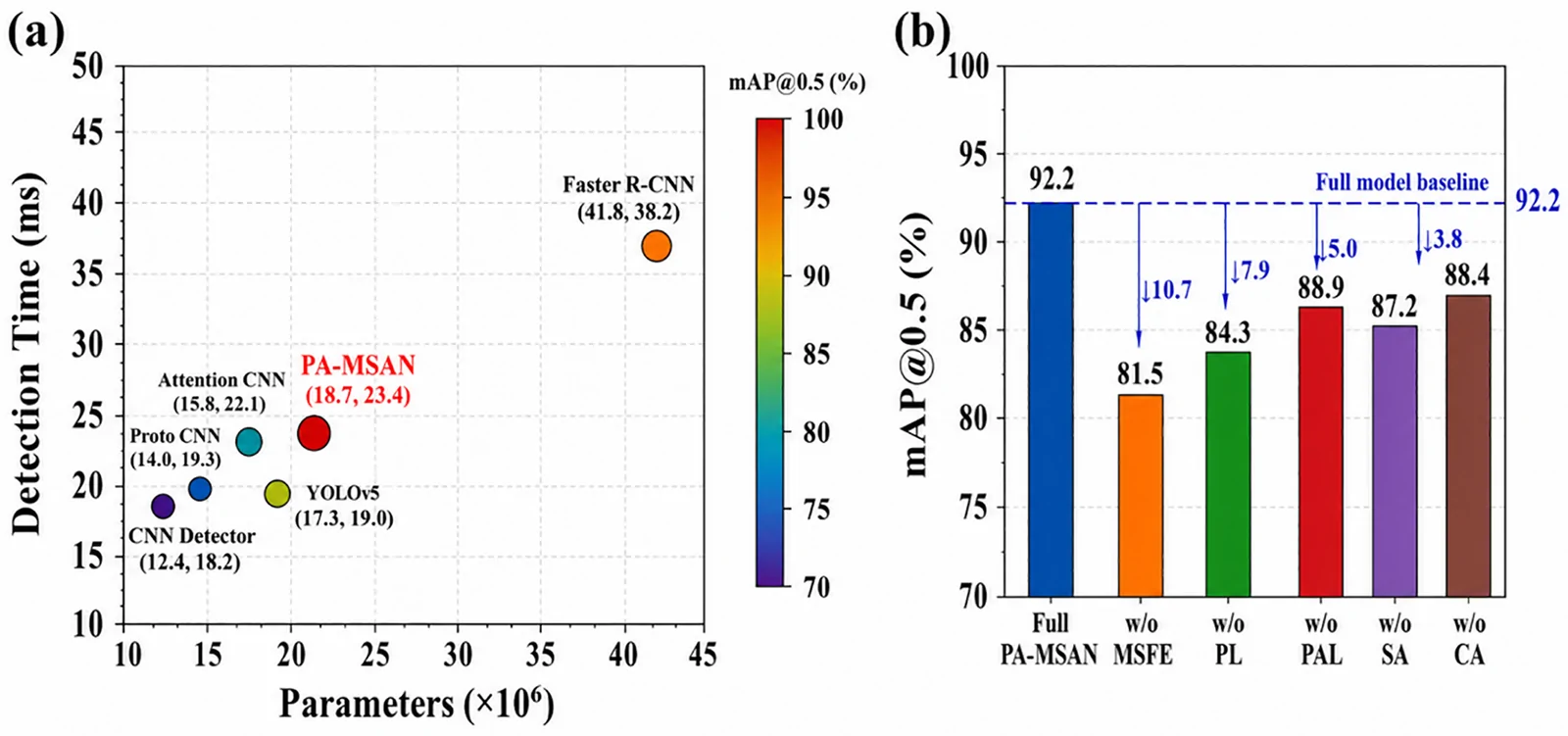
Overall performance and controlled ablation results. (a) Comparison of parameter count, detection time, and mAP@0.5. (b) mAP@0.5 under the full model and five ablation configurations.

Fig 14(a) compares parameter count, detection time, and mAP@0.5 for the evaluated methods. PA-MSAN reaches 92.2% mAP@0.5 with 18.7 × 10⁶ parameters and a detection time of 23.4 ms. Table 1 and Fig 14(b) summarize the controlled ablations: removing MSFE produces the largest mAP decrease (10.7 percentage points), removing the complete prototype-learning branch lowers mAP by 7.9 points, removing PAL alone lowers mAP by 3.3 points, and removing spatial or channel attention lowers mAP by 5.0 and 3.8 points, respectively. Table 4 separately evaluates the explicit multi-receptive-field configuration against the stacked-3 × 3 variant.

Attention placement is evaluated with three alternatives under the same 5-shot NEU-DET protocol: attention applied separately at each FPN level before fusion, attention applied after RoI Align, and attention applied only inside the RPN. Table 13 compares these variants with the proposed post-fusion, pre-RPN placement.

**Table 13:** Ablation of attention-module embedding positions under the 5-shot NEU-DET setting.

Variant	mAP@0.5 Mean ± Std (%)	mAP drop compared with full PA-MSAN (points)	Main interpretation
Proposed layout (dual attention after FPN fusion, before RPN)	92.2 ± 0.4	—	Highest mAP among the tested attention placements
Attention embedded per FPN level (pre-fusion)	90.8 ± 0.4	1.4	1.4-point lower mAP than the proposed placement
Attention applied after RoI Align (RoI-level)	91.3 ± 0.4	0.9	0.9-point lower mAP than the proposed placement
Attention inside RPN only	90.2 ± 0.5	2.0	2.0-point lower mAP than the proposed placement

Note: All variants share the same data split, random seeds 0–4, and training protocol as Table 1. The proposed layout corresponds to the full PA-MSAN model.

The post-fusion, pre-RPN placement reaches 92.2 ± 0.4% mAP@0.5, compared with 90.8 ± 0.4% for per-level pre-fusion attention, 91.3 ± 0.4% for post-RoI attention, and 90.2 ± 0.5% for RPN-only attention. Among the four evaluated placements, post-fusion pre-RPN attention achieves the highest mAP@0.5.

A stacked-3 × 3 variant is evaluated against the explicit 3 × 3/5 × 5/7 × 7 configuration using matched channel settings and the same FPN, attention modules, prototype branch, training budget, and evaluation protocol. The stacked-3 × 3 variant reaches 90.4 ± 0.4% mAP@0.5 and 85.9 ± 0.5% APsmall, while the proposed multi-kernel design reaches 92.2 ± 0.4% and 88.7 ± 0.4%, respectively. The corresponding feature-extractor parameter counts are 3.8 M and 4.0 M, and the reported FLOPs are 27.9 G and 25.7 G.

#### 4.3.3. Hyperparameter sensitivity analysis.

A sensitivity analysis evaluates the Prototype Alignment Loss weight $\lambda_{p}$ over {0.1, 0.3, 0.5, 0.8, 1.0}, with all other settings fixed and each configuration repeated for seeds 0–4. Table 9 reports 1-shot, 3-shot, and 5-shot mAP@0.5 together with the mean ± standard deviation of the best-validation epoch across repeated runs, and Fig 15 visualizes the corresponding trends.

**Fig 15:**
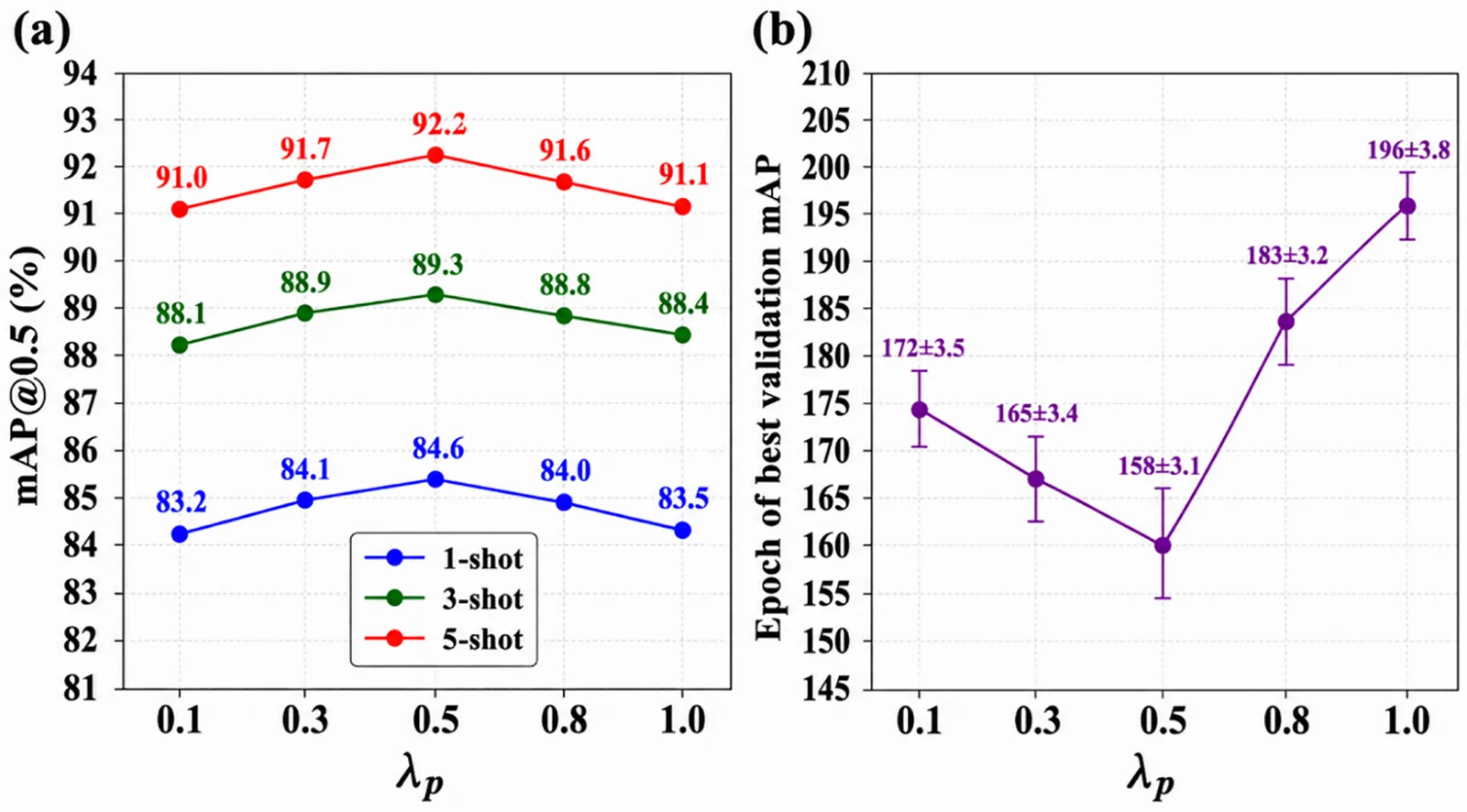
Sensitivity of detection performance and best-validation epoch to the Prototype Alignment Loss weight $\lambda_{p}$. (a) mAP@0.5 under 1-shot, 3-shot, and 5-shot settings for different $\lambda_{p}$ values. (b) Mean ± standard deviation of the best-validation epoch for different $\lambda_{p}$ values.

Table 9 shows that mAP@0.5 increases as $\lambda_{p}$ rises from 0.1 to 0.5 and decreases at 0.8 and 1.0. The $\lambda_{p}$ = 0.5 setting reaches 92.2 ± 0.4% in the 5-shot condition and 84.6 ± 0.3% in the 1-shot condition. Across seeds, the best-validation epoch is 158 ± 3.1 for $\lambda_{p}$ = 0.5, while larger weights reach their best validation scores later within the 200-epoch budget.

## 5. Discussion

The results characterize PA-MSAN under three coupled conditions in few-shot industrial surface inspection: limited labeled samples, substantial defect-scale variation, and background texture interference. The complete architecture combines multi-scale feature extraction, spatial-channel attention, and RoI-level prototype matching, and the ablations quantify the contribution of each component under the shared 5-shot protocol.

The multi-scale feature extractor combines feature-pyramid fusion with 3 × 3, 5 × 5, and 7 × 7 kernels to represent fine textures, intermediate defect structures, and broader spatial context. PA-MSAN reaches 88.7 ± 0.4% APsmall in the 5-shot NEU-DET setting, and removing MSFE lowers mAP@0.5 by 10.7 percentage points. The stacked-3 × 3 comparison in Table 4 further shows that the multi-kernel configuration yields higher mAP@0.5 and APsmall with comparable feature-extractor parameters and lower reported FLOPs.

Spatial-channel attention refines the fused feature representation before proposal generation. The complete model reaches 0.89 ± 0.01 average IoU and a 4.1 ± 0.2% background false response rate. Removing spatial attention lowers mAP@0.5 by 5.0 percentage points, and removing channel attention lowers it by 3.8 percentage points. These ablations are consistent with complementary roles for spatial localization and channel-wise feature selection in textured industrial backgrounds.

Prototype learning provides the few-shot class representation used by the final metric classifier. Support-region features are averaged into class prototypes and evaluation RoIs are compared with these prototypes by cosine similarity, while PAL regularizes the shared embedding. Removing the complete prototype-learning branch lowers mAP@0.5 by 7.9 percentage points, and removing PAL alone lowers it by 3.3 points. PA-MSAN also records higher 5-shot mAP@0.5 than TFA, Meta R-CNN, and FSRW under the shared evaluation protocol, indicating that the proposed RoI-level prototype formulation is competitive with the evaluated few-shot detectors on NEU-DET.

The general-purpose and few-shot-specific baselines provide complementary reference points. Under the 5-shot NEU-DET setting, PA-MSAN reaches 92.2 ± 0.4% mAP@0.5 with a reported detection time of 23.4 ms, compared with 87.6 ± 0.3% for YOLOv5, 89.3 ± 0.4% for Faster R-CNN, 88.4 ± 0.4% for TFA, 86.8 ± 0.5% for Meta R-CNN, and 85.9 ± 0.7% for FSRW. These results describe the accuracy-computation trade-off under the tested implementation and evaluation conditions.

Cross-domain evaluation assesses PA-MSAN under a change in dataset and target-class composition. With 5-shot GC10-DET support prototypes, the model reaches 86.4 ± 0.4% mAP@0.5, compared with 92.2 ± 0.4% on NEU-DET. The 5.8-point (6.3% relative) difference reflects the combined change in class semantics, class count, object scale, image distribution, and target-domain adaptation under limited labeled support.

The two-stage RPN/RoI Align configuration requires 23.4 ms per image and 18.7 × 10⁶ parameters on the reported platform. Structured pruning [46], knowledge distillation [47], and lightweight backbones such as MobileNetV3 [48] provide direct routes toward lower deployment cost. Evaluation on NEU-DET and GC10-DET covers steel and metallic surface defects; broader validation can extend the analysis to composite surfaces, semiconductor wafers, highly reflective components, and other production environments. Each class is represented by a single arithmetic-mean prototype, while adaptive support-feature correlation [43] and multi-prototype modeling [49] provide relevant directions for representing stronger intra-class morphological variation. Recent industrial defect research has highlighted information-density imbalance and heterogeneous local defect characteristics in practical surface inspection scenarios [50]. Self-supervised pre-training and parameter-efficient adaptation provide additional directions for improving data efficiency [51].

## 6. Conclusion

This study evaluates PA-MSAN for few-shot industrial surface defect detection with limited labeled samples, large defect-scale variation, and textured backgrounds. PA-MSAN combines multi-receptive-field feature-pyramid fusion, spatial-channel attention, RoI-level prototype matching, RPN proposal generation, and joint detection/prototype optimization. The evaluation covers controlled 1-shot, 3-shot, and 5-shot NEU-DET experiments together with limited-supervision cross-domain adaptation on GC10-DET.

The method constructs class prototypes from attention-enhanced support RoI features and uses cosine similarity for final few-shot classification. RPN and bounding-box regression optimize localization through the same RoI representation, while the auxiliary linear classification head and Prototype Alignment Loss provide classifier-space and metric-space supervision during training. The ablations separately evaluate multi-scale feature extraction, prototype learning, PAL, attention components, attention placement, and the explicit multi-kernel design.

Under the 5-shot NEU-DET protocol, PA-MSAN reaches 92.2 ± 0.4% mAP@0.5, 88.7 ± 0.4% APsmall, 0.89 ± 0.01 average IoU, and a 4.1 ± 0.2% background false response rate. Under the same evaluation protocol, it records higher mAP@0.5 than CNN Detector, Proto CNN, Attention CNN, YOLOv5, Faster R-CNN, TFA, Meta R-CNN, and FSRW. The controlled ablations show the largest mAP decrease after removing MSFE, followed by removal of the complete prototype-learning branch, spatial attention, channel attention, and PAL.

With 5-shot target-domain GC10-DET support, PA-MSAN reaches 86.4 ± 0.4% mAP@0.5, compared with 92.2 ± 0.4% on NEU-DET. The 5.8-percentage-point (6.3% relative) difference summarizes the cross-dataset result under limited target-domain supervision. The evaluation covers NEU-DET and GC10-DET with the two-stage PA-MSAN configuration. Future work will examine adaptive and multi-prototype aggregation, lighter deployment architectures, and broader validation across industrial materials and imaging conditions.

## Supporting information

S1 File Seed-level results for PA-MSAN and baseline methods on NEU-DET and GC10-DET. The file contains Table A, Table B, and Table C.
(DOCX)
