# Supplementary material for: A prototype-aligned multi-scale attention network for few-shot industrial surface defect detection
Source: PLoS One. 2026 Sep 28;21(9):e0359400. doi: 10.1371/journal.pone.0359400 (PMC13618933; doi:10.1371/journal.pone.0359400)
Supplement: Supplementary file 1 [file pone.0359400.s001.docx]

**S1 Supporting Information**

**Table A. Seed-level few-shot mAP@0.5 values of PA-MSAN on NEU-DET.**

| **Shot setting** | **Seed 0** | **Seed 1** | **Seed 2** | **Seed 3** | **Seed 4** | **Mean ± Std** |
| --- | --- | --- | --- | --- | --- | --- |
| 1-shot mAP@0.5 | 84.2 | 85.0 | 84.3 | 84.8 | 84.7 | 84.6 ± 0.3 |
| 3-shot mAP@0.5 | 88.9 | 89.5 | 89.1 | 89.7 | 89.3 | 89.3 ± 0.3 |
| 5-shot mAP@0.5 | 91.8 | 92.4 | 91.9 | 92.8 | 92.1 | 92.2 ± 0.4 |

**Table B. Seed-level 5-shot mAP@0.5 comparison with baseline methods on NEU-DET.**

| **Model** | **Seed 0** | **Seed 1** | **Seed 2** | **Seed 3** | **Seed 4** | **5-shot mAP@0.5** |
| --- | --- | --- | --- | --- | --- | --- |
| CNN Detector | 78.1 | 78.6 | 77.8 | 78.4 | 78.1 | 78.2 ± 0.3 |
| Proto CNN | 82.6 | 83.2 | 82.8 | 83.6 | 83.0 | 83.0 ± 0.4 |
| Attention CNN | 85.4 | 85.9 | 85.1 | 86.0 | 85.5 | 85.6 ± 0.4 |
| YOLOv5 | 87.3 | 87.8 | 87.2 | 88.0 | 87.7 | 87.6 ± 0.3 |
| Faster R-CNN | 88.9 | 89.5 | 88.8 | 89.8 | 89.3 | 89.3 ± 0.4 |
| TFA | 88.0 | 88.7 | 88.2 | 88.9 | 88.4 | 88.4 ± 0.4 |
| Meta R-CNN | 86.3 | 87.2 | 86.6 | 87.4 | 86.7 | 86.8 ± 0.5 |
| FSRW | 85.1 | 86.4 | 85.5 | 86.7 | 85.8 | 85.9 ± 0.7 |
| PA-MSAN | 91.8 | 92.4 | 91.9 | 92.8 | 92.1 | 92.2 ± 0.4 |

**Table C. Seed-level 5-shot mAP@0.5 comparison on GC10-DET.**

| **Model** | **Seed 0** | **Seed 1** | **Seed 2** | **Seed 3** | **Seed 4** | **5-shot mAP@0.5** |
| --- | --- | --- | --- | --- | --- | --- |
| CNN Detector | 64.0 | 64.7 | 64.3 | 65.1 | 64.4 | 64.5 ± 0.4 |
| Proto CNN | 71.2 | 71.9 | 71.4 | 72.3 | 71.7 | 71.7 ± 0.4 |
| Attention CNN | 76.5 | 77.2 | 76.8 | 77.5 | 77.0 | 77.0 ± 0.4 |
| YOLOv5 | 77.3 | 78.0 | 77.6 | 78.4 | 77.7 | 77.8 ± 0.4 |
| Faster R-CNN | 81.1 | 81.8 | 81.4 | 82.1 | 81.6 | 81.6 ± 0.4 |
| PA-MSAN | 86.0 | 86.7 | 86.2 | 86.9 | 86.2 | 86.4 ± 0.4 |
